# Activating PEG host to enable spatially controlled surface functionalization of nanocarriers for biomedical applications

**DOI:** 10.1126/sciadv.adu3932

**Published:** 2025-11-19

**Authors:** Mingchen Sun, Wei Li, Shaohua Zhang, Daniela A. Wilson

**Affiliations:** Institute for Molecules and Materials, Radboud University Nijmegen, Heyendaalseweg 135, 6525 AJ, Nijmegen, Netherlands.

## Abstract

Surface functionalization with biomolecules transforms nanocarriers by integrating a range of bioderived functionalities; however, conventional methods often yield low efficiency and uneven ligand distribution, and compromise the structural integrity of nanocarriers. Here, we introduce a breakthrough approach that activates the traditionally inert PEG corona, enabling precise and spatially controlled functionalization of nanocarriers with biologically relevant entities. Using fluorescence resonance energy transfer (FRET) between pyrene and FITC, we confirm accurate spatial distribution of ligands. Our method achieves exceptional efficiency and stability, maintaining over 40% of functional molecules across three cycles of washing and resuspension under various aqueous conditions. In vitro assays reveal high biological efficacy, with engineered polymersomes supporting targeted cellular interactions. Functionalization with diverse ligands introduces specific biological functionalities, including mitochondrial targeting, cell migration stimulation, and enhanced receptor-mediated endocytosis. This rapid, efficient, and user-friendly strategy for PEG surface functionalization heralds remarkable advances in nanomedicine and biomaterials.

## Teaser

Activating inert PEG corona enables controlled biomolecule functionalization for universal nanocarrier surface engineering.

## INTRODUCTION

Because of their important features, self-assembled nanocarriers hold great potential in various fields including drug delivery (*1*), diagnosis and imaging (*2*), vaccine development (*3*), and miniature medical devices (*4*). As of 2022, approximately 100 nanomedicine formulations had received approval from the US Food and Drug Administration and the European Medicines Agency (*5*). Despite this progress, ensuring nanocarriers reach their target site remains a critical challenge. Typically, biomolecules play a crucial role in directing nanoparticles specifically within biological environments, by leveraging biorecognition motifs, such as ligands or antibodies, or by incorporating bioderived functions such as catalysis into the nanoparticles. Currently, three primary strategies exist for engineering the surface of nanocarriers: (i) Conjugating functional ligands to the surface of preformed nanocarriers via noncovalent interactions (e.g., biotin-streptavidin binding, nitrilotriacetic acid–metal complexation, etc.) or covalent attachment (e.g., click chemistry); (ii) self-assembly of end-group functionalized block copolymers, where targeting ligands are grafted onto the end group of polymers, followed by mixing with unmodified polymers in a certain ratio; and (iii) incorporating biofunctional blocks such as glycopolymers or peptides directly into polymer backbones (*6*, *7*). However, these methods are often labor-intensive, with low efficiency that results in incomplete functionalization and reduced ligand availability. Covalent conjugation, which often involves reactive agents or organic solvents, can destabilize nanocarriers, induce cargo leakage, or alter nanocarrier properties (*8*). Furthermore, attaching targeting ligands to amphiphilic block copolymers may disrupt the hydrophilic/hydrophobic balance critical for nanocarrier self-assembly (*9*). In addition, during self-assembly, functional moieties in end-functionalized polymers can become buried or oriented inward, reducing their accessibility and biological activity (*10*). Therefore, a simple, spatially controlled and highly efficient functionalization strategy that overcomes these limitations is highly needed.

Although polyethylene glycol (PEG) has traditionally been regarded as a nonimmunogenic and inert polymer, our recent findings indicate that changes in thermal history can drive vesicles into distinct energy states through conformational changes that alter the interdigitation of oligo(ethylene glycol) chains. This modulation ultimately leads to the formation of diverse vesicular structures with complex energy profiles, which are anticipated to influence their functional properties (*11*). Furthermore, while PEG was historically considered highly hydrated and resistant to dehydration, both computational simulations and experimental studies have demonstrated that the release of water molecules from PEG can compensate for the entropy loss associated with chain conformational changes, thereby making PEG dehydration an energetically favorable process. Inspired by the induced-fit model of enzyme-substrate interactions, we proposed that PEG can dynamically adjust its conformation to accommodate the size and shape of guest molecules. Molecular dynamics simulations further supported this hypothesis and revealed that hydrophobic surfaces like graphene can trigger PEG dehydration, enhancing its interaction with guest molecules (*12*, *13*). Among various polycyclic aromatic hydrocarbons, pyrene demonstrated remarkable affinity and loading capacity for the PEG corona. Isothermal calorimetric titration and molecular dynamics simulations not only affirmed the thermodynamic favorability of this process (Δ*H* < 0 and Δ*S* > 0) but also highlighted its rapid nature (around 10 ns) (*14*, *15*). Starting from these groundbreaking discoveries, we propose a “mix and match” strategy to efficiently activate the PEG corona for advanced nanocarrier surface engineering using the insertion of biorelevant pyrene constructs onto the PEG corona. Our approach maintains the nanocarrier’s morphology unaffected while maximizing ligand exposure on the surface, thus enabling an efficient platform for spatially controlled surface functionalization of nanocarriers.

**Fig. 1.**
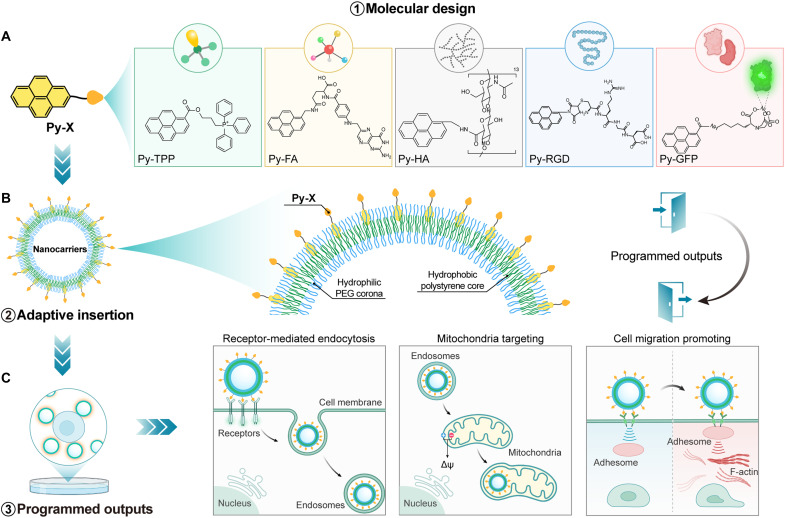
Spatially controlled surface functionalization by activating the PEG host for biomedical applications. (**A**) Chemical structures of Py-Xs representing different classes of bioactive compounds. (**B**) High-density and spatially -controlled surface functionalization of PEG_44_-b-PS_167_ polymersomes. The membrane of the polymersomes consists of a hydrophobic polystyrene core and two hydrophilic PEG coronas. (**C**) Schematics of various biological events triggered by Py-Xs–functionalized polymersomes: receptor-mediated endocytosis, mitochondrial targeting, and cell migration promoting.

To validate our hypothesis, we devised and synthesized five pyrene-conjugated molecules spanning important and biologically-relevant molecular categories and investigated their interaction with the PEG shell (Fig. 1, A and B). Using micrometer-sized polymersomes made of poly(ethylene oxide)-*b*-poly(1,2-butadiene) (PEG_44_-*b*-PBD_180_), we visualized the binding process with confocal microscopy. Besides, fluorescein isothiocyanate (FITC) was incorporated as the fluorescence resonance energy transfer (FRET) acceptor to further validate the colocalization of Py-Xs with the polymeric membrane. Subsequently, we assessed the changes in the zeta potential and the stability of polymersomes in different aqueous conditions. By calculating the number of Py-Xs on each polymersome, we quantified the efficiency and density of our functionalization strategy. Last, we conducted a series of in vitro experiments to confirm the robustness and effectiveness of our noncovalent binding strategy (Fig. 1C). We envisioned that our strategy provides an efficient, rapid, and user-friendly platform for the surface functionalization of polymeric vesicles and other PEGylated nanocarriers, thereby opening an avenue for the development of drug delivery carriers, biomaterials, and nanomedicine.

## RESULTS

### Molecular design

To assess the applicability and versatility of this approach, we selected one representative molecule from a diverse set of compounds typically challenging to attach to nanocarriers, including small organic molecules, vitamins, polysaccharides, peptides, and proteins (Fig. 1). Each chosen molecule also possesses specific, well-recognized biological functions, providing a robust test of the method’s effectiveness. As a representative small organic molecule, we selected triphenylphosphine (TPP). Mitochondria, vital organelles in eukaryotic cells, are integral to cellular energy metabolism and the regulation of programmed cell death (*16*). Dysfunctions or malfunctions of mitochondria have been associated with various diseases, including cancer, characterized by enhanced anabolism, unlimited proliferation potential, and impaired autophagy (*17*, *18*). Consequently, there is increasing interest in strategies aimed at targeting mitochondria for therapeutic interventions. TPP accumulates in mitochondria due to the negative membrane potential generated by mitochondrial respiratory complexes (*19*–*21*). Folic acid (FA), frequently used for targeting cells overexpressing the folate receptor (FR), was selected for its role in tumor targeting and endocytosis. The high prevalence of FR overexpression in human tumors makes it an ideal marker for targeting delivery and cancer imaging (*22*). Upon binding with its ligand, clathrin is recruited and assembled on the cell membrane and subsequently forms the clathrin-coated pits and endosomes for endocytosis (*23*). Therefore, we coupled FA with pyrene and investigated the endocytosis of Py-FA–functionalized nanocarriers. Hyaluronic acid (HA) is a linear polysaccharide widely distributed in the eye vitreous, connective tissues, joints, etc. Its binding to the extracellular domain of CD44 promotes interactions with multiple cytoskeletal proteins (ankyrin, RhoGTPases, etc.), then regulates the cytoskeletal dynamics and cell motility (*24*). As a result, HA has been widely studied in targeting CD44 and tumor migration (*25*). Therefore, we selected HA as the representative polysaccharide for this study. To further investigate the applicability and effectiveness of peptides and proteins, we coupled the RGD peptide to pyrene via a thiol-maleimide reaction. The recognition and binding of RGD with integrin receptors triggers a conformational change in the integrin receptor, resulting in endocytosis through multiple pathways such as clathrin-mediated endocytosis, caveolin-mediated endocytosis, clathrin- and caveolin-independent pathways, etc. As a proof of concept, green fluorescent protein (GFP) was chosen as a model protein. We coupled GFP and pyrene through the specific binding of nitrilotriacetic acid–nickel complexation (NTA-Ni2^+^) and oligohistidine sequences of GFP. The fluorescence of GFP makes it possible to observe its attachment to the PEG surface. The critical micelle concentrations of all molecules were measured using the changes in I_1_/I_3_ in the fluorescence emission of Py-Xs and are presented in fig. S3.

### Insertion of Py-Xs onto PEG corona

#### Confirmation of Py-Xs loading onto PEG corona

**Fig. 2.**
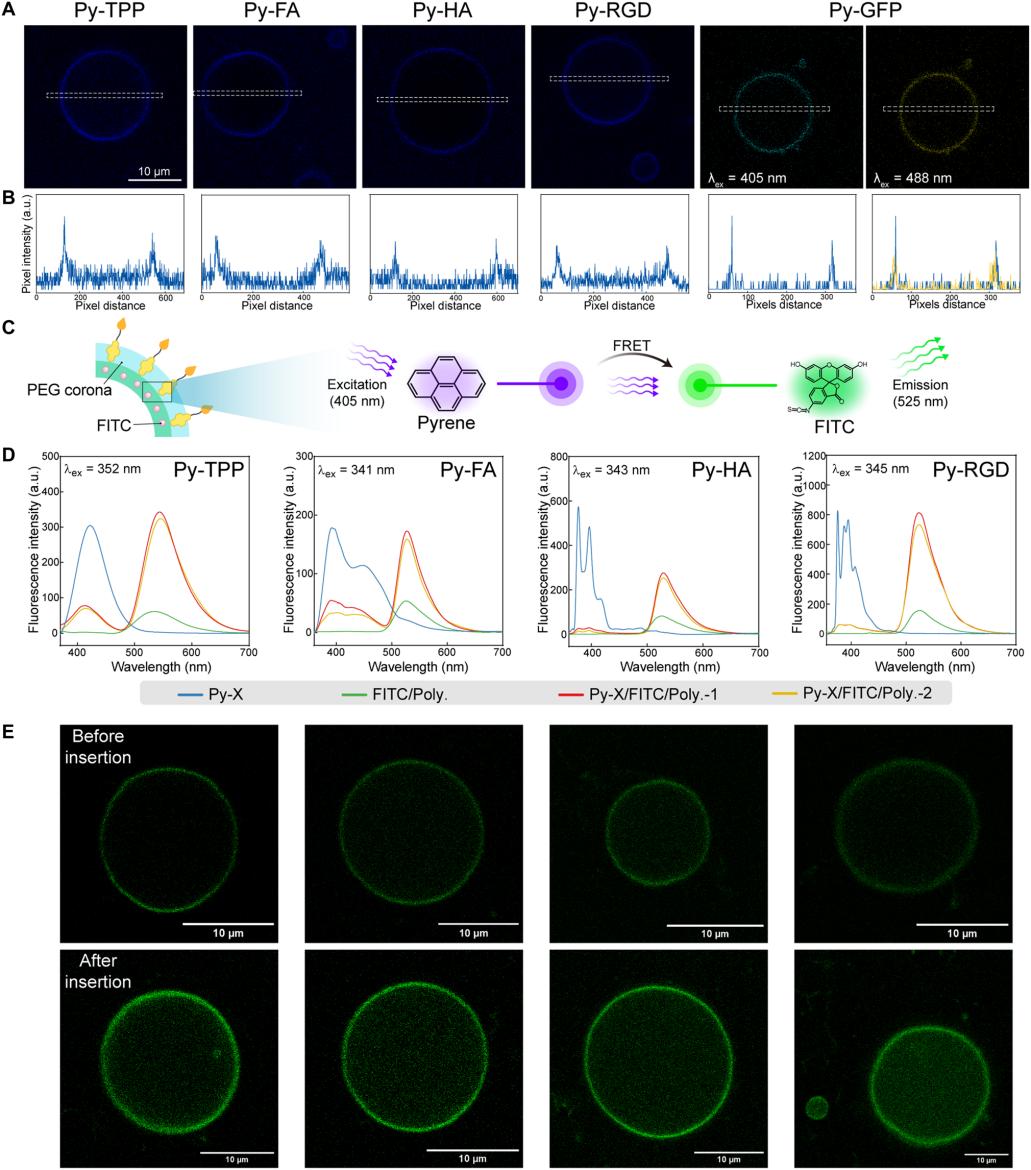
Visualization of Py-Xs inserting onto PEG corona. (**A**) Fluorescence images of micrometer-sized polymersomes loaded with Py-Xs and the corresponding plots (**B**) showing the fluorescence intensity along the marked areas (pyrene: λ_ex_ = 405 nm, λ_em_ = 435 nm; GFP: λ_ex_ = 488 nm, λ_em_ = 520 nm). (**C**) Schematic showing the fluorescence resonance energy transfer (FRET) between pyrene and FITC. (**D**) Fluorescent emission spectra of Py-X, FITC-labeled polymersomes (FITC/Poly.), FITC-labeled polymersomes loaded with Py-X (Py-X/FITC/Poly.-1), and FITC-labeled polymersomes loaded with Py-X after centrifugation (Py-X/FITC/Poly.-2). (**E**) Fluorescence images of micrometer-sized polymersomes loaded with or without Py-Xs (λ_ex_ = 405 nm, λ_em_ = 520 nm).

To visualize the insertion of Py-Xs onto the PEG corona, we adopted the co-block polymer PEG_950_-*b*-PBD_2000_, which can self-assemble to form micrometer-sized polymersomes with PEG corona. In our previous study, we demonstrated that pyrene approaches the PEG corona and forms van der Waals interactions within 10 ns, then remains confined in a binding pocket characterized by high PEG density and low water content (*14*, *15*). When Py-Xs are inserted onto the PEG corona, Py-Xs excited by a laser at a certain wavelength emit fluorescence, making the micrometer-sized polymersomes visible under confocal microscopy. As shown in Fig. 2A, micrometer-sized polymersomes loaded with Py-Xs (Py-TPP, Py-FA, Py-HA, and Py-RGD) showed clear fluorescence. Fluorescence intensity along the marked area suggested that the fluorescence was evenly distributed across the membrane (Fig. 2B). As for Py-GFP loaded polymersomes, we separate excitations at 405 and 488 nm to obtain the fluorescence signal of pyrene and GFP, respectively. The fluorescence intensity plots showed a notable coincidence between the fluorescence signals from the two channels, suggesting that both pyrene and GFP were colocalized on the polymeric membrane.

Next, we used FRET to further validate the insertion behavior of Py-Xs. FITC was chosen as the acceptor due to its lipophilic nature, which allows for labeling the membrane, as well as its spectral overlap with pyrene (Fig. 2C). As shown in Fig. 2D, upon excitation at 340 to 350 nm, Py-Xs exhibited strong emission spectra while FITC-labeled polymersomes (FITC/Poly.) showed minimal fluorescence. However, when Py-Xs were mixed with the polymersome suspension, the fluorescence of FITC markedly intensified, indicating successful energy transfer from pyrene to FITC. To rule out any interaction between Py-Xs in the solvent and FITC-labeled polymersomes, we centrifuged the system and resuspended it in water before repeating the measurement. As depicted by the light yellow lines, the fluorescence intensities of Py-X after centrifugation (Py-X/FITC/Poly.-2) remained consistent with those before centrifugation (Py-X/FITC/Poly.-1, orange lines), thereby confirming the insertion of Py-Xs onto the polymersomes. Subsequently, we used confocal microscopy to monitor this process. As shown in Fig. 2E, the FITC incorporated in the bilayers of micrometer-sized polymersomes was not fully excited under the laser at 405 nm. In contrast, the fluorescence intensities of FITC-labeled polymersomes increased notably due to the energy transfer from pyrene to FITC. The quantitative analysis of the fluorescence images confirmed this trend (fig. S4).

To demonstrate the broader applicability of our strategy, we prepared ultrasmall polymeric nanovesicles (sPoly, ~189 nm, fig. S6), PEG nanogels (~162.5 nm, fig. S7), and PEGylated liposomes (~180 nm, fig. S8), and evaluated their functionalization with Py-Xs. Using dSTORM-TIRF imaging, we visualized distinct fluorescence clusters corresponding to each nanoparticle type, confirming successful ligand insertion below the diffraction limit. Loading stability assays showed that all five ligands maintained comparable retention to that of larger polymersomes, with sPoly and nanogels retaining >45% and >65% of Py-Xs, respectively, and liposomes maintaining >60% insertion after repeated centrifugation. Py-FA/sPoly demonstrated effective and enhanced endocytosis in HeLa cells, indicating preserved ligand bioactivity. Together, these results confirm that our strategy enables robust and stable surface functionalization of high-curvature sub–200 nm nanocarriers, nonbrush PEG nanoparticles, and clinically relevant PEGylated lipid nanoparticles, remarkedly expanding the scope and translational potential of our approach.

#### Investigation of loading stability

**Fig. 3.**
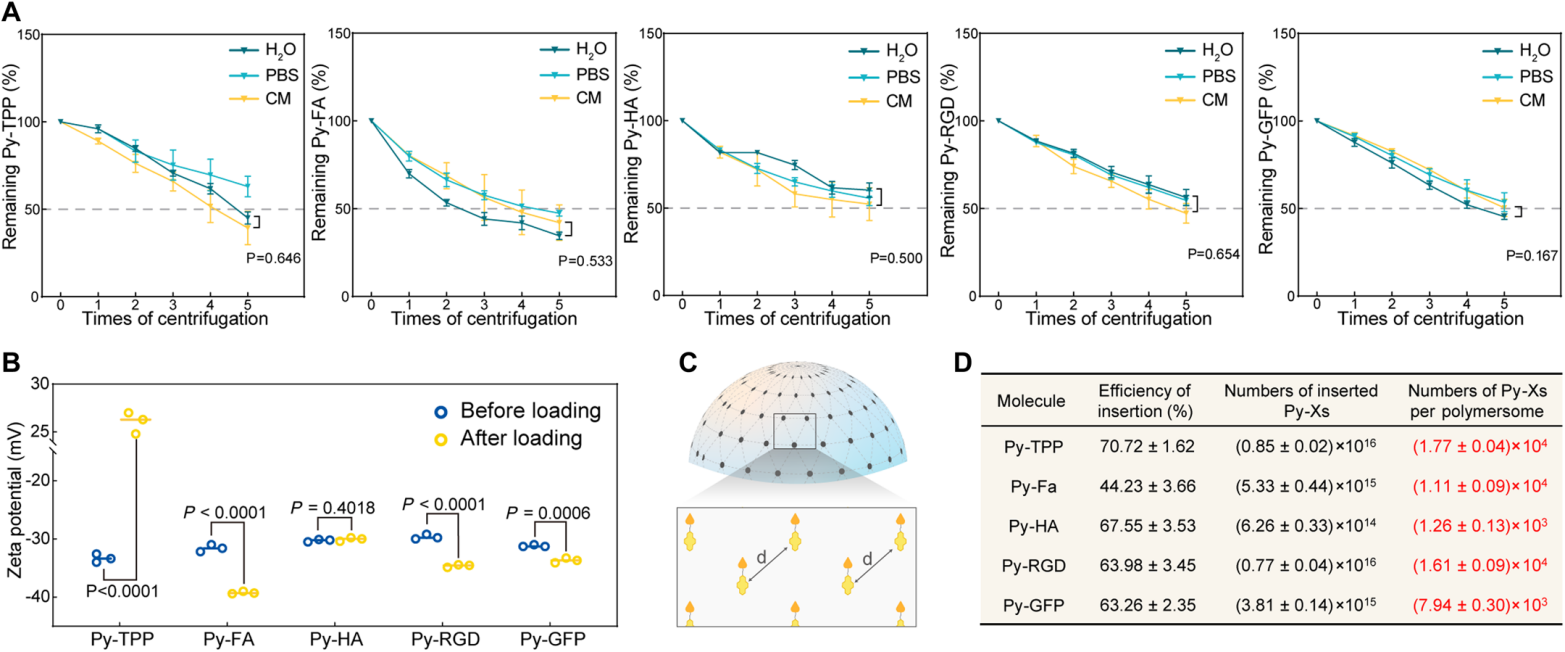
Investigation of stability and efficiency. (**A**) Stability test of Py-Xs loading onto PEG_44_-b-PS_167_ polymersomes in three different aqueous conditions: Milli-Q water, phosphate-buffered saline (PBS, pH 7.2), and complete medium for cell culture (CM). Centrifugation: 14,000 rpm, 10 min. (**B**) Zeta potential of PEG_44_-b-PS_167_ polymersomes before and after the loading of Py-Xs. (**C**) Schematic depicting high-density surface functionalization by the insertion of Py-Xs. (**D**) Quantitative results of adaptive insertion after three rounds of centrifugation (*N*_A_ = 6.02 × 10^23^).

As a previously unexplored method for polymersome functionalization, the stability of the system holds considerable importance. In our study, we used sequential centrifugation and resuspension steps to investigate the loading stability of each molecule (Fig. 3A). During centrifugation, varying degrees of detachment of Py-Xs were observed. After five cycles of centrifuging and resuspending, Py-TPP showed better stability in phosphate-buffered saline (PBS) compared to milli-Q water, with around 63% of Py-Xs remaining inserted (45.03 ± 3.59% versus 63.01 ± 5.93%, Milli-Q versus PBS, *P* = 0.01). Overall, after five centrifugation cycles, over 40% of Py-Xs remained inserted. To simulate the physiological environment, we also evaluated loading efficiency in a complete medium for cell culture [CM; phenol red-free Dulbecco’s Modified Eagle Medium (DMEM) supplemented with 10% of fetal bovine serum (FBS), penicillin (100 U/ml), and streptomycin (100 μg/ml). No significant difference was found in the loading of Py-X between Milli-Q water and the CM (Fig. 3A and table S3). The insertion of Py-TPP, Py-FA, and Py-RGD generated notable changes in the zeta potential of the nanosized polymersomes (Fig. 3B and table S1). The zeta potential of polymersomes after Py-TPP loading increased from −33.33 ± 0.70 to 26.03 ± 1.12 and remained stable after multiple centrifugation cycles. In contrast, unreacted carboxyl groups in Py-FA and Py-RGD further reduced the surface potential of the functionalized polymersomes (Fig. 3B) (−31.60 ± 0.60 versus −39.00 ± 0.21, mV versus mV, before Py-FA loading versus after Py-FA loading, *P* < 0.0001; −29.67 ± 0.42 versus −34.63 ± 0.23, mV versus mV, before Py-RGD loading versus after Py-RGD loading, *P* < 0.0001).

#### Quantification

Drawing inspiration from the mechanism through which viruses invade cells by attaching viral spikes to host cell receptors, numerous nanocarriers have been designed to display specific moieties, since many cellular processes are driven by antigen-receptor recognition and binding (*26*). Hence, the quantity and distribution of ligands on the surface of nanocarriers remarkably affect their biological properties (*27*). In this part, we determined the number of Py-X molecules inserted per polymersome by measuring the concentration of Py-Xs remaining in the supernatant after each centrifugation step using ultraviolet-visible (UV-vis) spectroscopy. The polymersome concentration was quantified by Nanosight LM10. The number of inserted Py-Xs per polymersome (*n*) was calculated using

$$n=\frac{C_{m}\times N_{A}}{C_{p}\times N_{A}\times k}$$

Where *C*_m_ is the measured concentration of Py-Xs in the supernatant, *C*_p_ is the polymersome concentration, *N*_A_ is Avogadro’s number, and *k* is the dilution factor of the polymersome suspension. As shown in Fig. 3 (C and D), after three rounds of centrifugation, approximately 1.77 × 10^4^, 1.11 × 10^4^, and 1.16 × 10^4^ Py-TPP, Py-FA, and Py-RGD remained inserted per polymersome. Because of the relatively large size and steric bulk of the HA molecule, the number of Py-HA molecules was found to be 1.26 × 10^3^. In the case of GFP, a protein consisting of 238 amino acids with a molecular mass of 27 kDa (*28*), a smaller number of Py-GFP inserted onto peg corona was expected. As a result, one polymersome was functionalized by around 7.94 × 10^3^ GFP molecules. More quantitative data about the stability are provided in table S2.

### Programmed biological functions

#### Mitochondria targeting facilitated by Py-TPP

**Fig. 4.**
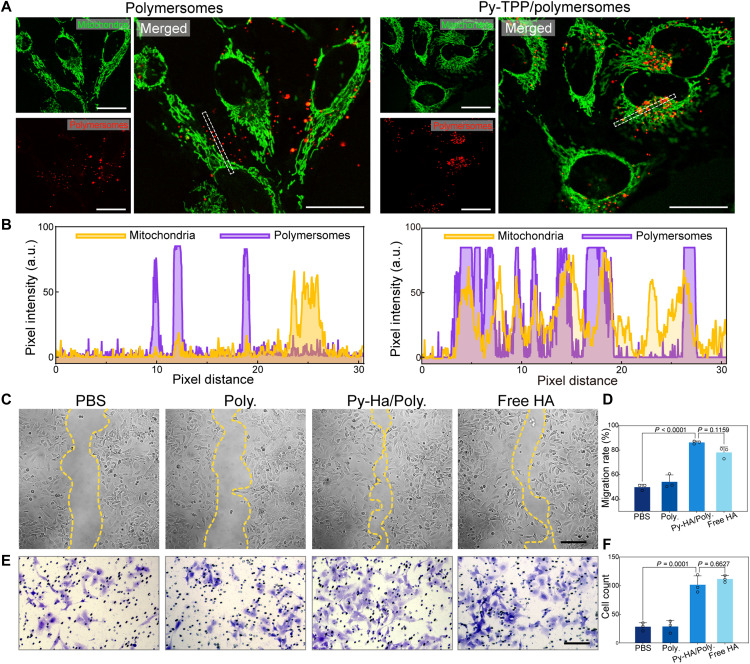
Mitochondrial targeting facilitated by Py-TPP– and Py-HA–functionalized polymersomes promote SKOV-3 cell migration. (**A**) Fluorescence images of HeLa cells incubated with polymersomes and with Py-TPP–functionalized polymersomes. (mitochondria: λ_ex_ = 490 nm, λ_em_ = 523 nm; Nile red–labeled polymersomes: λ_ex_ = 560 nm, λ_em_ = 635 nm; scale bars, 20 μm). (**B**) Fluorescence intensities of mitochondria and polymersomes along the marked area analyzed by ImageJ. (**C**) Microscopy images showing the migration of SKOV-3 cells after 12 hours of different treatments (scale bars = 50 μm). (**D**) Semiquantitative analysis of migration rate of SKOV-3 cells by ImageJ. (**E**) Cell migration assay using a transwell insert at 48 hours (scale bars = 100 μm). (**F**) Cell counting of migrated SKOV-3 cells under different treatments.

Among many mitochondrial targeting strategies, delocalized lipophilic cations have been demonstrated to accumulate in mitochondria due to the highly negatively charged microenvironment of the mitochondrial matrix (*20*). TPP, in particular, has received considerable attention for this purpose. To verify whether the insertion of Py-TPP onto the surface of polymersomes preserved their mitochondrial -targeting capacity, we incubated Py-TPP–functionalized polymersomes with HeLa cells. As shown in Fig. 4A, upon 6 hours of incubation, the red fluorescence of Nile red–labeled polymersomes and the green fluorescence of mitochondrial fluorescent dye were distributed separately. However, following treatment with Py-TPP–functionalized polymersomes, the two fluorescence signals overlapped, indicating successful targeting of the polymersomes to mitochondria. This colocalization was further confirmed by intensity profiles of the two fluorescence signals along the marked area (Fig. 4B).

#### Cell migration promoted by Py-HA

CD44 is a hyaluronan receptor that is highly expressed in many cancer cells. Recent studies have suggested that CD44 modulates the secretion and activation of matrix metalloproteinase-9 (MMP-9). The proteolytically active MMP-9 plays a crucial role in collagen IV degradation and promotes tumor cell invasion. Therefore, we performed the cell scratch assay to investigate the migration capacity of SKOV-3 cells incubated with Py-HA–functionalized polymersomes. As shown in Fig. 4 (C and D), cells incubated with Py-HA–functionalized polymersomes (Py-HA/Poly.) showed a significantly higher migration rate than cells treated with PBS within 12 hours (49.77 ± 2.32% versus 86.32 ± 1.13%, PBS versus Py-HA/Poly., *P* < 0.0001). In addition, no significant difference was found between the migration ratios of Py-HA/Poly. and free HA (50 μg/ml) (86.32 ± 1.13% versus 78.14 ± 4.48% and PBS versus free HA, *P* = 0.1159). We further validated the migration-enhancing effect using a transwell assay. As shown in Fig. 4E, the incubation with Py-HA/Poly. and free HA resulted in notable enhancements in the number of migrated cells, with notable differences being found between the PBS group and the Py-HA/Poly group (Fig. 4F).

#### Endocytosis mediated by Py-FA

**Fig. 5.**
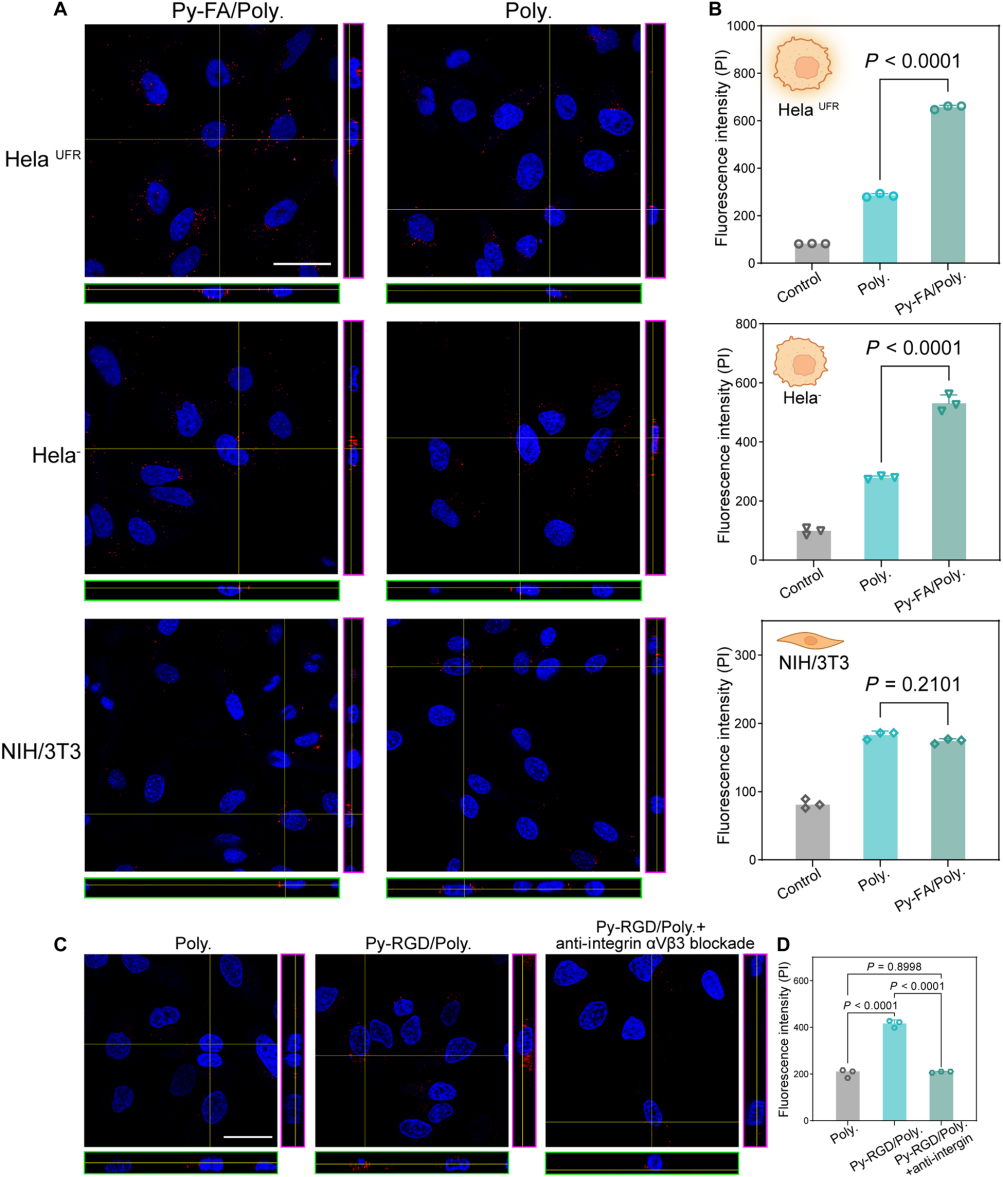
Functionalization with Py-FA and Py-RGD enhances endocytosis. (**A**) Orthogonal views of fluorescence images showing HeLa^UFR^, HeLa^−^, and NIH/3T3 cells incubated with polymersomes and Py-FA–functionalized polymersomes (scale bars = 20 μm). (**B**) Flow cytometry analysis of polymersomes and polymersomes functionalized with Py-FA uptake by HeLa^UFR^, HeLa^−^, and NIH/3T3 cells. The control group represents untreated cells. (**C**) Orthogonal views of fluorescence images showing HeLa cells incubated with polymersomes and polymersomes loaded with Py-RGD (scale bars = 20 μm). (**D**) Flow cytometry analysis of cells incubated with polymersomes, polymersomes loaded with Py-RGD, and anti-integrin αVβ3 (10 μg/ml) plus polymersomes loaded with Py-RGD.

FR is a membrane receptor overexpressed in many types of tumor cells. The targeting of FR has demonstrated effectiveness in facilitating the uptake of drugs and nanocarriers (*29*). Here, we cultured normal HeLa cells (HeLa^−^) in an FA-deficient medium to induce up-regulation of FR expression, resulting in HeLa cells with up-regulated (HeLa^UFR^) (*30*). Mouse embryonic fibroblasts, NIH/3T3 cells were used as a negative control as they lack FR expression (*31*). As displayed in Fig. 5 (A and B), after incubating with Py-FA–functionalized polymersomes (Py-FA/Poly.) or unfunctionalized polymersomes (Poly.) for 6 hours, both HeLa^UFR^ and HeLa^−^ exhibited higher uptake of Py-FA/Poly. compared to Poly. Specifically, HeLa^UFR^ cells internalized approximately 2.3 times more Py-FA/Poly. than Poly. (285.3 ± 7.7 versus 657.0 ± 8.7, Poly. versus Py-FA/Poly., *P* < 0.0001), while for HeLa^−^, this increase was 1.89-fold (279.0 ± 6.0 versus 530.7 ± 29.3, Poly. versus Py-FA/Poly., *P* < 0.0001). This enhancement is attributed to the higher FR expression in HeLa^UFR^ cells, which facilitated great cellular uptake. In contrast, NIH/3T3 cells showed no notable difference in the uptake of Py-FA/Poly. and Pol, further supporting the role of FR-mediated endocytosis (182.7 ± 5.7 versus 174.0 ± 3.6, Py-FA/Poly. versus Poly., *P* = 0.21). In the case of unfunctionalized polymersomes, HeLa^UFR^ and HeLa^−^ internalized similar amounts of polymersomes, confirming that FA functionalization on polymersome surfaces specifically enhances FR-mediated uptake.

#### Endocytosis mediated by Py-RGD

As a representative member of the integrin family, the integrin αvβ3 receptor has been proven to be associated with various types of cancer, such as melanoma, glioblastoma, and breast cancer. Therefore, the surface functionalization of nanocarriers with ligands targeting integrin αvβ3 holds promise as a potential approach for targeted cancer therapy. In this part, we investigated the endocytosis behavior of Py-RGD–functionalized polymersomes. Figure 5C illustrates a notable difference in the uptake of polymersomes (Poly.) compared to Py-RGD–functionalized polymersomes (Py-RGD/Poly.) by HeLa cells. Furthermore, when integrin αvβ3 was blocked using its specific antibody, the endocytosis of Py-RGD/Poly decreased to a level equivalent to that of nonfunctionalized polymersomes (Poly.), suggesting that the enhanced endocytosis was specifically mediated by the RGD-integrin interaction. This trend was quantitatively validated by flow cytometry (Fig. 5D).

## DISCUSSION

Traditional covalent conjugation techniques, such as carbodiimide chemistry and click chemistry, involve the formation of stable covalent bonds between ligands and nanoparticle surfaces. While these methods ensure strong ligand attachment, they often require complex synthesis procedures, harsh reaction conditions, and can potentially alter the bioactivity of the ligands (*32*). In addition, covalent modifications may introduce challenges related to reproducibility and scalability in functionalizing nanocarriers (*33*). Noncovalent approaches, including electrostatic interactions, hydrophobic interactions, and hydrogen bonding, offer an alternative to covalent methods. However, these noncovalent interactions can be sensitive to environmental factors such as pH and ionic strength, potentially leading to reduced stability and inconsistent functionalization outcomes (*34*–*36*). By leveraging the strong affinity between pyrene and the PEG corona of the nanocarriers, our method facilitates the stable and efficient attachment of bioactive molecules. Compared to traditional covalent methods, our approach simplifies the functionalization process, eliminating the need for complex chemical reactions and preserving ligand integrity. Unlike other noncovalent methods that may suffer from environmental sensitivities, the pyrene-PEG interaction provides enhanced stability, ensuring consistent performance in diverse biological settings. This versatility enables the engineering of polymersomes with tailored biological functionalities, such as mitochondrial targeting, promotion of cell migration, and enhanced endocytosis. Moreover, our study demonstrates the robustness of the functionality of the engineered polymersomes, with ligands maintaining their bioactivity, allowing for spatially controlled ligand distribution, and showing excellent performance across various aqueous environments, including water, phosphate buffer, and cell culture medium. We envision this strategy as an innovative, simple, and user-friendly method for the surface functionalization of nanocarriers in drug delivery, diagnostic, imaging, and vaccine development.

## MATERIALS AND METHODS

### Loading of Py-Xs onto micrometer-sized polymersomes

Microsized polymersomes with PEG corona were prepared using PEG_950_-*b*-PBD_2000_ by the hydration method. Specifically, 1.25 mg of PEG-*b*-PBD was dissolved in 1 ml of chloroform, and the organic solvent was removed by a direct stream of nitrogen. After desiccation in a vacuum for 4 hours, the giant polymeric vehicle was formed upon the addition of 3 ml of water and incubation at 60°C for 48 hours. Micrometer-sized polymersome suspensions were then mixed with Py-Xs aqueous solutions (final concentrations, 20 μM). After incubating for 10 min, images of microsized polymersomes were captured by a confocal microscope (λ_ex_ = 405 nm and λ_em_ = 465 nm). For Py-GFP, fluorescent images were obtained using the 4′,6-diamidino-2-phenylindole (DAPI) channel (λ_ex_ = 405 nm and λ_em_ = 465 nm) and GFP channel (λ_ex_ = 488 nm and λ_em_ = 510 nm) separately. ImageJ was used for the analysis of radical fluorescence intensity.

### Fluorescence resonance energy transfer

The fluorescence excitation spectrums and emission spectrums of Py-Xs are presented in fig. S2. FITC-labeled PEG_44_-b-PS_167_ polymersomes (FITC/Poly.) were prepared according to the above-mentioned method except for adding 40-μl FITC solution [dissolved in tetrahydrofuran (THF), 10 mg/ml] before self-assembly. Free FITC was removed by repeated centrifugation (14,000 rpm, 10 min). The emission spectra of Py-Xs solutions, FITC/Poly. suspension, and the mixture of Py-Xs and FITC/Poly. were determined by a fluorescence spectrometer at the optimal excitation wavelength of each molecule. FITC-labeled giant polymeric vehicles were prepared by adding 5 μl of FITC solution (10 mg/ml in THF) to the PEG-*b*-PBD solution before desiccation. Free FITC was removed by dialysis (molecular weight cutoff = 12,000 to 14,000 Da) against Milli-Q water for 48 hours. After incubating with Py-Xs (final concentrations, 20 μM), fluorescent images were obtained using the DAPI channel (λ_ex_ = 405 nm and λ_em_ = 465 nm) and GFP channel (λ_ex_ = 488 nm and λ_em_ = 510 nm) separately.

### Preparation of nanometer-sized polymersomes

PEG_44_-b-PS_167_ (10 mg) was dissolved in a 1-ml mixture of distilled THF and dioxane (4:1, v/v) in a 15-ml vial with a magnetic stirring bar. After 30-min stirring, 0.5-ml Milli-Q water was added via a syringe pump at a rate of 1 ml/hour while stirring vigorously. Approximately 10 ml of Milli-Q was added to quench the polymersomes. Repeated centrifugation (14,000 rpm, 10 min) was used to remove the organic solvent.

### Loading stability and efficiency

The quantitative methodology of each Py-Xs was established by plotting the linear curve of Py-Xs and corresponding UV absorption values (fig. S1). PEG_44_-b-PS_167_ polymersomes were resuspended in Milli-Q water, PBS buffer, and cell culture medium [phenol red–free DMEM supplemented with 10% of FBS, penicillin (100 U/ml), and streptomycin (100 μg/ml)], followed by mixing with Py-Xs (final concentration, 20 μM). During repeated centrifugation and resuspending, the concentrations of Py-Xs in the supernatants after each centrifugation were detected by a UV-vis spectrometer. The concentration of polymersomes in PEG_44_-b-PS_167_ polymersome suspension, as well as resuspended suspensions, was quantified by Nanosight LM10.

### Zeta potential

One microliter of polymersome suspension containing 1 mg of polymer was mixed with Py-Xs and incubated for 10 min. The final concentrations of Py-Xs were 20 μM. Afterward, the sediment obtained by centrifugation (14,000 rpm, 10 min) was repeatedly resuspended with Milli-Q water, and the ζ-potential of each suspension was detected by dynamic light scattering.

### Cell culture

All cells were purchased from the American Type Culture Collection. HeLa cells and NIH/3T3 cells were cultured in DMEM. SKOV-3 cells were cultured in RPMI 1640 medium. All mediums contain 10% FBS and antibiotics [penicillin (100 U/ml) and streptomycin (100 μg/ml)] at 37°C with 5% CO_2_. Trypsin/EDTA was used to digest cells.

### Mitochondrial targeting

HeLa cells were seeded in an eight-well plate (Ibidi GmbH) with a density of 5 × 10^4^ per well and incubated overnight for adherence. Py-TPP–modified polymersomes labeled with Nile red (Py-TPP/NR/Poly., 150 μg/ml) were incubated with HeLa cells at 37°C for 6 hours. After PBS rinsing (three times), green mitochondria dye staining (100 nM, 20 min), and formaldehyde fixation (4% paraformaldehyde, 15 min), fluorescent images were obtained by confocal fluorescence microscopy. The colocalization of mitochondria and polymersomes was analyzed with ImageJ software (NIH). Polymersomes labeled with Nile red (NR/Poly.) were used as control.

### Endocytosis mediated by FR

HeLa cells were cultured in folate acid–deficient medium (RPMI 1640 Medium, no FA, Thermo Fisher Scientific) for 10 passages to obtain HeLa cells with up-regulated FR expression (HeLa^UFR^). HeLa^UFR^, normal HeLa cells (HeLa^−^), and NIH/3T3 cells were seeded in 12-well plates with a density of 5 × 10^4^ cells/ml. After incubation overnight, Py-FA–modified polymersomes labeled with Nile red (Py-Fa/NR/Poly.) and polymersomes labeled with Nile red (NR/Poly.) were added to each well with a final concentration of 300 mg/ml, followed by incubation at 37°C for 6 hours. After PBS rinsing, and DAPI (1 μg/ml) staining, fluorescent images were captured by confocal fluorescence microscopy. ImageJ was used to count the number of polymersomes taken up by cells.

### Cell migration promoted by Py-HA

SKOV-3 cells were seeded to culture-inserts (Culture-Insert 2 Well in μ-Dishm, Ibidi GmbH) at a density of 3 × 10^4^ cells/ml. After 24 hours, the inserts were removed and cells were left in the treatment media (PBS, Poly. Py-Fa/Poly., and free HA) for another 12 hours of incubation. Cell migration was evaluated by microscopy and ImageJ was used to analyze the scratched areas. Anti-CD44 (10 μg/ml) was used as a control. For the transwell-based migration assay, SKOV-3 cell suspension at a density of 1 × 10^6^/ml was added to the upper chamber of transwell inserts and was incubated at 37°C, 5% CO_2_ for 30 min. Afterward, the transwell inserts were inserted into an eight-well plate filled with cell culture media containing PBS, Poly. Py-Fa/Poly., and free HA. After 48 hours, cells on the upper side of the insert were removed, and cells on the bottom side were fixed [4% PFA for 15 min at room temperature (RT)] and stained with 0.2% crystal violet for 20 min at RT. An Echo Revolution microscope was used to observe the cell migration.

### Endocytosis mediated by integrin αvβ3

HeLa cells were seeded in eight-well plates with a density of 5 × 10^4^ cells per well. After overnight incubation, Py-RGD–modified polymersomes labeled with Nile red (Py-RGD/NR/Poly.) and polymersomes labeled with Nile red (NR/Poly.) were added to each well with a final concentration of 300 mg/ml, followed by incubation at 37°C for 6 hours, After PBS rinsing and DAPI staining, fluorescent images were captured by confocal fluorescence microscopy. ImageJ was used to count the number of polymersomes uptake by cells. Cells treated with 10 μg/ml of anti-integrin αvβ3 were used as a control.

### Statistical analysis

All quantitative experiments were analyzed for statistical significance between the sampled conditions. The statistical tests used [Student’s *t* test for two groups, one-way analysis of variance (ANOVA) test, and Tukey’s post hoc test for more than two groups] are defined in the corresponding figure legends for all panels. Statistical significance is shown as follows: 0.01 < *P < 0.05, 0.001 < **P < 0.01, ***P < 0.001, and NS = not significant (*P* > 0.05).

## Supplementary Materials

### The PDF file includes:

Supplementary Text Figs. S1 to S8 Tables S1 to S3 Legend for data S1 References
