# Supplementary material for: Activating PEG host to enable spatially controlled surface functionalization of nanocarriers for biomedical applications
Source: Sci Adv. 2025 Nov 19;11(47):eadu3932. doi: 10.1126/sciadv.adu3932 (PMC12629181; doi:10.1126/sciadv.adu3932)
Supplement: Supplementary file 1 — Supplementary Text Figs. S1 to S8 Tables S1 to S3 Legend for data S1 References [file sciadv.adu3932_sm.pdf]

Science Advances
AAAS

Supplementary Materials for

Activating PEG host to enable spatially controlled surface functionalization of nanocarriers for biomedical applications

Mingchen Sun *et al.*

Corresponding author: Daniela A. Wilson, d.wilson@science.ru.nl

Sci. Adv. **11**, eadu3932 (2025)
DOI: 10.1126/sciadv.adu3932

The PDF file includes:

Supplementary Text
Figs. S1 to S8
Tables S1 to S3
Legend for data S1
References

Other Supplementary Material for this manuscript includes the following:

Data S1

Supplementary Text

Chemicals and materials

1-Pyrenecarboxylic acid, potassium carbonate (K_2CO_3), 3-bromopropyl triphenylphosphonium bromide, folic acid, N-(3-dimethylaminopropyl)-N′-ethylcarbodiimide hydrochloride (EDC·HCl) N-hydroxysuccinimide (NHS), and 1-pyrenemethylamine were purchased from Sigma-Aldrich. Hyaluronic acid (MW 5k) was purchased from Creative PEGworks. Nickel(ii) Chloride was purchased from TCI Europe NV. SH-RGD peptide (RGD) was purchased from Genscript Biotech. Recombinant Green Fluorescent Protein (GFP) was purchased from Biomatik Corporation. Poly(ethylene oxide)-b-poly(1,2-butadiene) (PEG_{22}-*b*-PBD_{37}, PDI=1.01) was purchased from Polymer Source Inc. Nile red was purchased from Fisher Scientific. Fluorescein isothiocyanate (FITC), Cell Counting Kit-8 (CCK-8), BioTracker^{TM} 488 Green Mitochondria Dye, anti-Integrin αVβ3, and anti-CD44 were purchased from Sigma-Aldrich. ATTO 488 NHS-ester was purchased from ATTO-TEC GmbH. PEGDA(MW=2k) and Irgacure 2959 were purchased from Sigma-Aldrich. DOTAP, DSPE-PEG_{2000} and cholesterol were purchased from TCI Europe NV . Water used in all experiments was deionized water produced by the Millipore instrument. All reagents were used as received without purification unless otherwise indicated.

Instruments and characterizations

^{1}H-NMR. Proton nuclear magnetic resonance (^{1}H-NMR) spectra were collected on a Bruker 400 MHz Avance III HD nanobay spectrometer with a BBFO probe. Tetramethylsilane (δ = 0.0 ppm), the residual protons of the deuterated solvent or dimethyl sulfone (δ = 3.0 ppm) were used as an internal reference. ^{1}H-NMR spectra were acquired using 32 scans and a relaxation delay of 5 s.

DLS. Dynamic light scattering (DLS) measurements were carried out at 25°C using Malvern Zetasizer Nano-ZS (Malvern Instruments) equipped with a He-Ne laser (633 nm, 4 mW) and an Avalanche photodiode detector (173°) to evaluate 0 the average hydrodynamic diameter (D_h) and polydispersity (PDI) of polymersomes. DLS uses the fluctuations in scattered light intensity due to the Brownian movement of the particles to determine their size distribution derived from the Stokes-Einstein equation by assuming a hard-sphere model.

Confocal microscopy. Confocal imaging was performed on a Leica (Wetzlar, Germany) SP8x AOBS-WLL confocal laser scanning microscopy with 40×, 63×, or 10× objectives (553-800 nm) at 25°C. Bidirectional scan direction X and a scan speed of 1000 Hz were adopted. The analysis of a fluorescence intensity profile was performed with NIH Image J.

Quantification of Py-Xs by UV spectroscopy

The quantitative detection methodology of each Py-X was established by plotting the linear curve of concentration and UV absorption. Specifically, precisely prepared the solutions of Py-Xs in series of concentrations (Py-TPP: 45.5, 18.2, 9.1, 4.6, and 1.8 µM; Py-FA: 20.0, 10.0, 5.0, 2.0, 1.0 µM; Py-HA: 20.0, 10.0, 5.0, 2.0, 1.0 µM; Py-RGD: 25.0, 10.0, 5.0, 2.5, 1.0 µM, Py-NTA: 20.0, 10.0, 5.0, 2.5, 1.0 µM) with Milli-Q water. Their UV absorption spectrums were obtained by a UV spectrophotometer. Quantitative linear curves were obtained by plotting the concentrations and the UV absorption versus the corresponding concentrations.

Quantitative analysis

The aqueous solutions of Py-Xs were mixed with 1 mL of PEG-PS polymersomes suspension. The final concentrations of Py-Xs were 20 µM. During repeated centrifugation and resuspending,

the concentration of Py-Xs in the supernatant after each centrifugation was detected by a UV-vis spectrometer. The concentration of polymersomes (C_p) in PEG-PS polymersomes suspension, as well as resuspended suspensions, was measured by Nanosight LM10. C_m is the concentration of molecules in supernatants obtained above the number of molecules inserted on each polymersome (n) was calculated by the following formula:

$$n = \frac{C_m \times N_A}{C_p \times N_A \times k}$$

Where N_A = 6.02 $\times 10^{23}$ and k is the dilution ratio of the polymersomes suspension during measurement.

Separation distance between molecules. Because of the electrostatic repulsion, TPP, and FA should be homogenously distributed on the surface of the polymersomes. The separation distance between adjacent molecules (d) was calculated as follows:

$$d = \sqrt{\frac{\pi D^2}{n}}$$

Where D is the diameter of polymersomes, determined to be 437.48 ± 3.27 nm by DLS. n was the number of molecules inserted on each polymersome after three rounds of centrifugation. In conclusion, for Py-TPP, d was calculated to be 5.83 ± 0.06 nm, which means each molecule occupies 33.95 ± 0.72 nm^2. In the case of Py-FA, d was calculated to be 7.36 ± 0.08 nm, and an area of 54.14 ± 1.14 nm^2 is taken up by one Py-FA molecule.

Critical micelle concentrations of Py-Xs

Precisely prepare solutions of Py-TPP, Py-FA, Py-HA, Py-RGD, and Py-NTA at a series of concentrations, followed by sonicating in an ultrasonication bath (VMR ultrasonic cleaner, 200 W) for 5 min to completely dissolve the molecule or to obtain a homogenous colloid. Afterward, the fluorescence emission spectrums were obtained at corresponding excitation wavelengths. Among peaks in the fluorescent spectrum of pyrene, the intensity ratio between the peak at 373 nm and 383 nm (I_1/I_3) is affected by the polarity of the surrounding solvent media. Therefore, the concentration where I_1/I_3 showed a notable decrease was assigned to be the critical micelle concentration (CMC) of Py-Xs.

Stability in fluids

To evaluate the stability of Py-X insertion under physiological fluids, we performed stability tests in a dynamic fluidic environment using a cell culture medium. Briefly, the upper and bottom receiving channels of the µ-Slide III 3in1 (3 mm Channel, ibidi) were filled with cell culture medium containing 10% serum, while polymersomes inserted with Py-Xs were suspended in the same medium and injected through the middle receiving channel. To mimic the shear stress found in venules (~15 dyn/cm^2) (37), the total flow rate across all receiving channels was set to 9.16 mL/min. The experiment was conducted at 37°C for 10 minutes. Afterwards, all liquid phases were centrifuged (14000 rpm, 10 min) to collect the polymersomes. UV-vis spectrometry was then used to analyze the stability of the insertion of each Py-X. The stability of Py-Xs insertions is shown in the table below.

Quantification of micrometer-sized polymersomes

The quantification of the fluorescence intensity of micrometer-sized polymersomes was performed with the software ImageJ. Six areas on the polymeric membrane were selected. The fluorescence intensity was corrected by the following formula (CTCF, corrected total cell fluorescence):

$$CTCF = Intergated\ Density - (Area\ selected \times Mean\ backgroundfluorescence\ )$$

Py-FA and Py-RGD enhance endocytosis detected by flow cytometry

$Hela^{UFR}$, $Hela^{-}$, and NIH/3T3 cells were seeded in 6-well plates with a density of 5×10^{4} cells/mL. After incubation overnight, Py-FA-modified polymersomes and Py-RGD functionalized polymersomes labeled with Nile red (NR/Poly.) were added to each well with a final concentration of 300 mg/mL, followed by incubation at 37°C for 6 h, After PBS rinsing and fixing by 4% PFA, the fluorescence intensity of each cells were detected by flow cytometry using the propidium iodide (PI) channel.

Insertion of Py-Xs onto small polymersomes

Preparation of small polymersomes (sPoly). Small polymersomes were prepared similarly to the 400 nm polymersomes. Briefly, PEG_{44}-b-PS_{167} (10 mg) was dissolved in a 1 mL mixture of distilled THF and dioxane (4:1, v/v) in a 15 mL vial with a magnetic stirring bar. After 30 min stirring, 0.5 mL Milli-Q water was added via a syringe pump at a rate of 1 mL/h while stirring vigorously. Upon completion of the addition, the mixture was filtered three times through a 220 nm syringe filter. Approximately 10 mL of Milli-Q was then added to quench the polymersomes. Repeated centrifugation (14000 rpm, 10 min) was used to remove the organic solvent. A JEOL TEM 1400 microscope at an acceleration voltage of 120 kV and DLS were used to characterize their morphology and diameter (38).

Single-molecule imaging of sPoly. Py-ATTO 488 was synthesized via the amide reaction between 1-pyrenecarboxylic acid and ATTO 488 NHS-ester. 50 µL of sPoly suspension (5 mg/mL) was mixed with 50 µL dSTORM imaging buffer and transferred to an 8-well dish with glass bottom (µ-Slide 8 Well high Glass Bottom, ibidi) for 30 min. After adding 10 µL of Py-ATTO (1 mg/mL), super-resolution microscopy images of Py-ATTO-labelled polymersomes were captured using an ONI Nanoimager (Oxford Nanoimaging) equipped with a 100×, 1.4 numerical aperture oil immersion objective and NimOS software. A total of 5000 frames were recorded using the 488 nm laser for fluorescence mapping. Fluorescence images were analyzed using CODI online platform (https://alto.codi.bio/).

Loading efficiency, stability, and endocytosis. The loading efficiency and stability of Py-Xs on sPoly., as well as the endocytosis into HeLa cells, were assessed following the same protocols described for the 400 nm polymersomes.

Insertion validation on PEG nanogels

Preparation of PEG nanogels. 50 µL of PEGDA water solution (30% in Milli-Q water, w/w) containing 1% Irgacure 2959 (w/w) was gently added to 400 µL of N-hexadecane without disturbing the liquid phases, followed by vortexing for 180 s. The resulting hydrogel droplets were cured by exposure to a focused UV beam (λ= 300-600 nm, 50% intensity, 140 mW/cm^{2}) for 5 min. Afterwards, the emulsion was centrifuged and washed with ethanol and water, respectively. DLS and a JEOL TEM 2100 cryo-electron microscopy at an acceleration voltage of 200 kV were used for the characterization.

Single-molecule imaging of PEG nanogels. Similar to the imaging of sPoly, 50 µL of PEG hydrogel suspension (0.3 mg/mL) was mixed with 50 µL dSTORM imaging buffer and transferred to an 8-well dish with glass bottom (µ-Slide 8 Well high Glass Bottom, ibidi) for 30 min. After adding 10 µL of Py-ATTO (1 mg/mL), super-resolution microscopy images of Py-ATTO-labelled PEG nanogels were captured following the same protocol described above.
Loading efficiency and stability. The loading efficiency and stability of Py-Xs were assessed following the same protocols described for the 400 nm polymersomes.

Insertion validation on lipid nanoparticles (LNP)

Preparation of lipid nanoparticles. DOPTA and cholesterol (1:1 molar ratio) were dissolved in dichloromethane. A thin lipid film was formed by removing the organic solvent using a rotary evaporator. Then, the lipid film was hydrated with 1 mL of Milli-Q water to achieve a final concentration of 10 mmol/L for both DOTAP and cholesterol. The liposome suspension was extruded through a 220 nm syringe filter 10 times. Next, 10 µL of DSPE-PEG (10 mg/mL) was further added, and the whole system was incubated at 55 °C for 15 min. DLS and a JEOL TEM 2100 cryo-electron microscopy at an acceleration voltage of 200 kV were used for the characterization (39, 40).
Single-molecule imaging of LNP. Similar to the imaging of sPoly, 50 µL of LNP (10 mmol/L) was mixed with 50 µL dSTORM imaging buffer and transferred to an 8-well dish with glass bottom (µ-Slide 8 Well high Glass Bottom, ibidi) for 30 min. After adding 10 µL of Py-ATTO (1 mg/mL), super-resolution microscopy images of Py-ATTO-labelled LNP were captured following the same protocol described above.
Loading efficiency and stability. The loading efficiency and stability of LNP were assessed following the same protocols described for the 400 nm polymersomes.

Synthesis

Synthesis of Py-TPP

1-Pyrenecarboxylic acid (74 mg, 0.3 mmol) and K_2CO_3 (55 mg, 0.4 mmol) were dissolved in 3 mL of THF and stirred at room temperature for 30 minutes under N_2. 3-Bromopropyl triphenylphosphonium bromide (97 mg, 0.2 mmol) was added to the reaction mixture and stirred overnight under N_2 at room temperature. The crude product was purified by column chromatography on silica gel (heptane: ethyl acetate: ethanol = 20:56:24) (107 mg, 67%).

^{1}H NMR (400 MHz, $CDCl_3$) δ 9.18 (d, *J* = 9.4 Hz, 1H), 8.61 (d, *J* = 8.1 Hz, 1H), 8.31 – 8.06 (m, 7H), 7.87 (ddd, *J* = 12.7, 8.3, 1.3 Hz, 6H), 7.73 (td, *J* = 7.4, 1.8 Hz, 3H), 7.62 (td, *J* = 7.7, 3.4 Hz, 6H), 4.87 (t, *J* = 6.2 Hz, 2H), 4.33 – 4.22 (m, 2H), 2.31 (d, *J* = 7.6 Hz, 2H). Py-TPP: MS (ESI): calculated for [C38H30O2P]+: m/z 549.20, found: m/z 549.14 $[M+H]^+$.

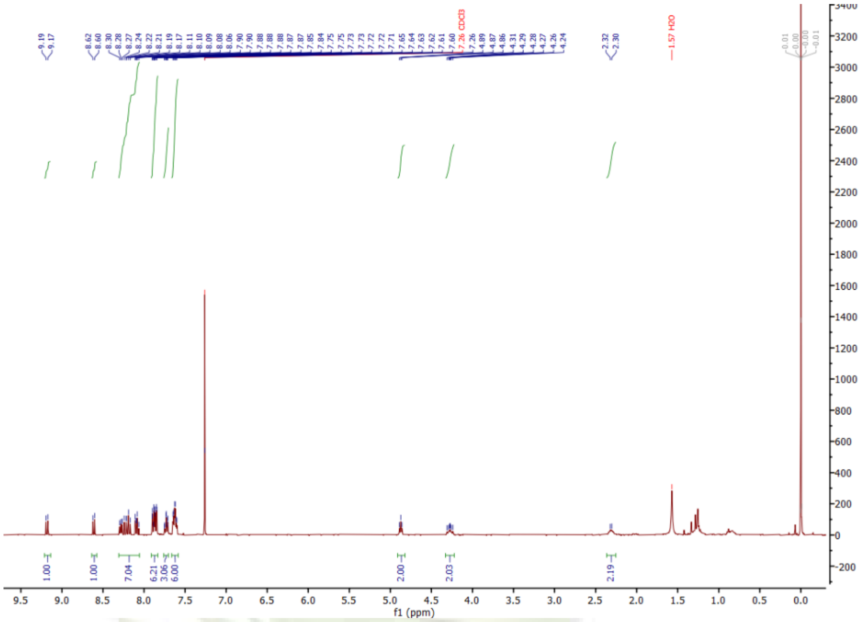

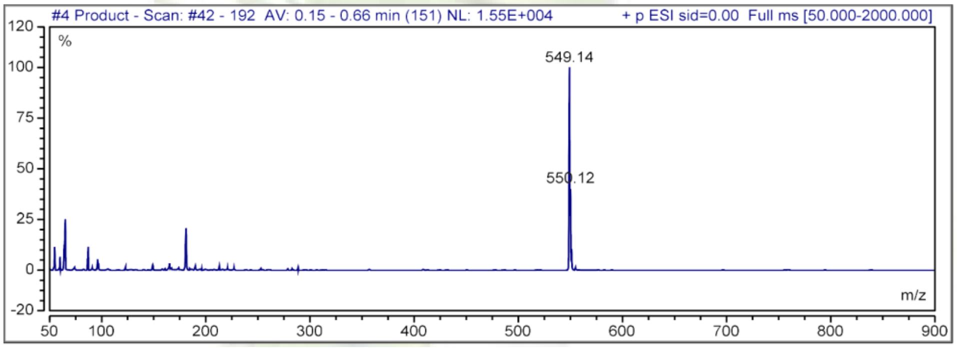

Synthesis of Py-FA

Folic acid (88 mg, 0.2 mmol) was dissolved in 2 mL of anhydrous dimethylformamide (DMF), followed by the addition of N-(3-Dimethylaminopropyl)-N′-ethylcarbodiimide hydrochloride (EDC·HCl, 37.2 mg, 0.24 mmol) and N-Hydroxysuccinimide (NHS, 46 mg, 0.4 mmol). The reaction mixture was stirred for 30 minutes at 40°C under N_2. 1-Pyrenemethylamine (46 mg, 0.2 mmol, 1 eq) was added, and the mixture was heated at 50°C overnight. The reaction mixture was precipitated with 30 mL of acetonitrile and the resulting yellow precipitate was washed three times with diethyl ether and Milli-Q water, respectively (62.9 mg, 48%).

^{1}H NMR (400 MHz, DMSO) δ 8.66 – 8.62 (m, 1H), 8.61 – 8.49 (m, 1H), 8.38 – 7.96 (m, 8H), 7.71 – 7.62 (m, 2H), 6.92 (d, J = 4.3 Hz, 1H), 6.67 – 6.60 (m, 2H), 5.00 (dd, J = 12.6, 7.1 Hz, 2H), 4.47 (t, J = 5.8 Hz, 2H), 4.31 (q, J = 7.4 Hz, 1H), 2.34 – 2.26 (m, 2H), 2.06 – 1.86 (m, 2H), 1.24 (s, 2H). Py-FA: MS (ESI): calculated for $[C36H30O8N5]^+$: m/z 654.23, found: 654.09 $[M+H]^+$, m/z 672.68 $[M+H_2O+H]^+$.

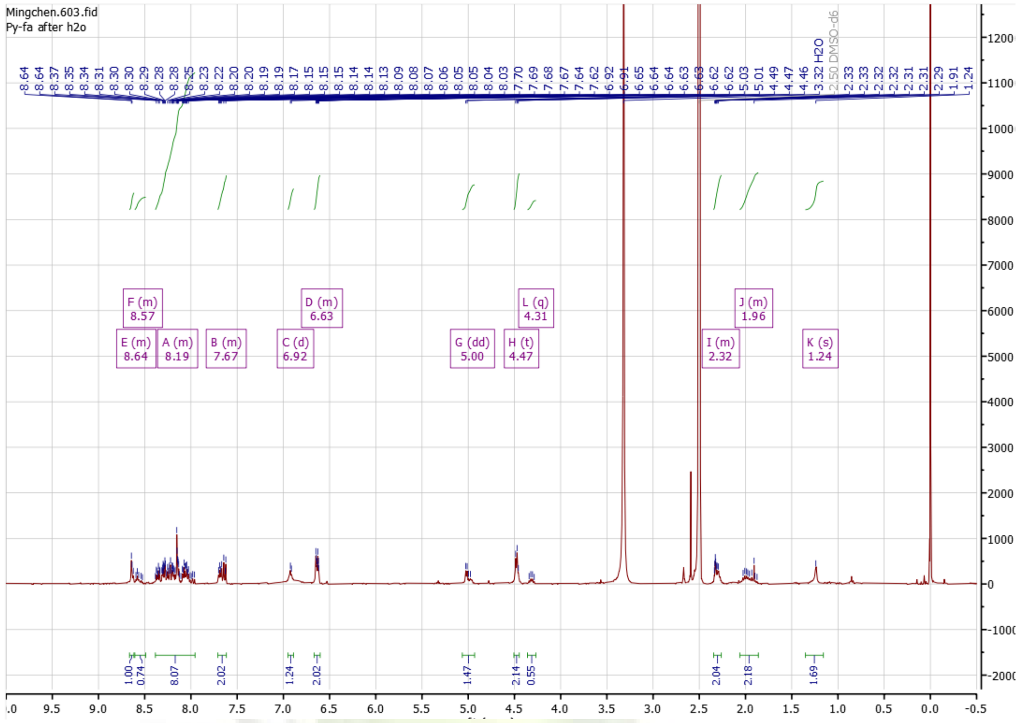

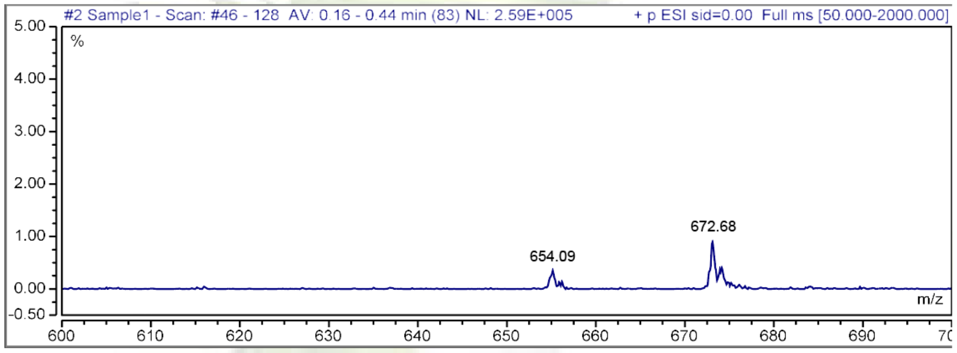

Synthesis of Py-HA

Hyaluronic acid (HA, MW=5000, 150 mg, 0.36 mmol of carboxyl group, 0.03 mmol of HA, 1 eq), 1-Pyrenemethylamine (313 mg, 1.17 mmol. 3 eq), EDC·HCl (225 mg, 1.17 mmol, 3 eq), and NHS (254 mg, 1.17 mmol, 3 eq) was dissolved in a 20 mL mixture of DMOS and water (1:1, v/v). The pH of the reaction was adjusted to 7 by the addition of 0.1 M NaOH and the reaction was proceed overnight at 30°C. The crude product was purified by washing with methanol and water three times, respectively (107 mg, 67%). ^{1}H NMR (500 MHz, DMSO) δ 8.51 – 7.99 (m, 9H), 1.96 – 1.66 (m, 3H).

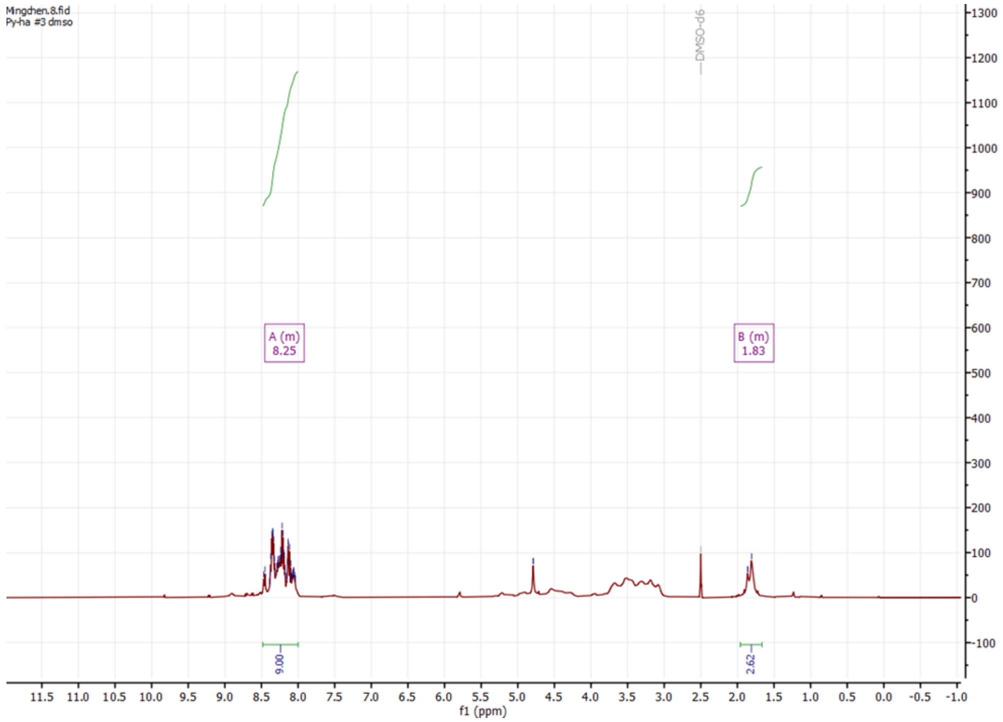

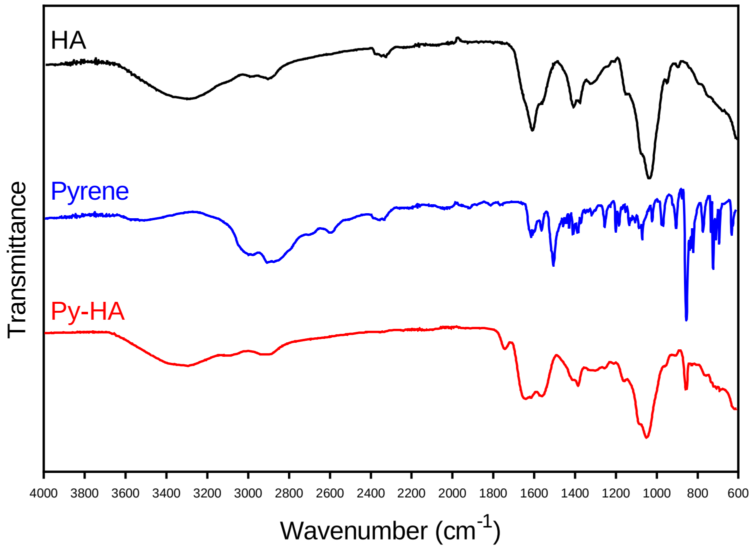

Synthesis of Py-RGD

CRGD (45 mg, 0.1 mmol) and N-(1-Pyrenyl)maleimide (14.85 mg, 0.05 mmol) were dissolved in 2 mL degassed Milli-Q water and 2 mL degassed anhydrous DMSO, respectively. The CRGD solution was added dropwise to the N-(1-Pyrenyl)maleimide solution using a syringe pump (4 mL/h) under vigorous stirring at room temperature. The resulting solution was stirred for an additional 2 hours before purification. Py-RGD was purified using a Shimadzu LC-20 RP-HPLC system equipped with a Phenomenex C18 column and detected using differential refractive index or UV absorbance (254 nm). Elution conditions: 0-80% ACN in MilliQ over 20 min; 80-

100% ACN in MilliQ over 2 min, 100% ACN in MilliQ for 3 min; 100-0% ACN in MilliQ over 3 min (16.4 mg, 44%). The product was characterized by AccuTOF-CS. MS (ESI): calculated for [C38H38N8O9S]+: m/z 746.25, found: m/z 747.09 $[M+H]^{+}$, m/z 374.2 $[M+2H]^{2+}$.

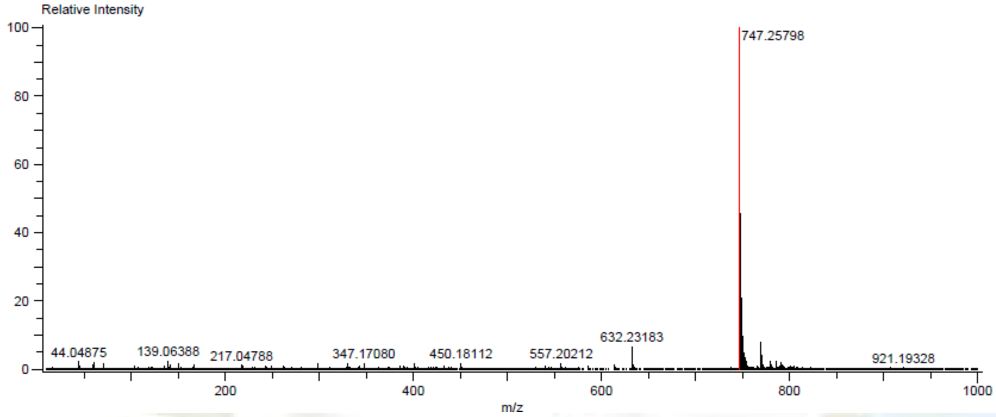

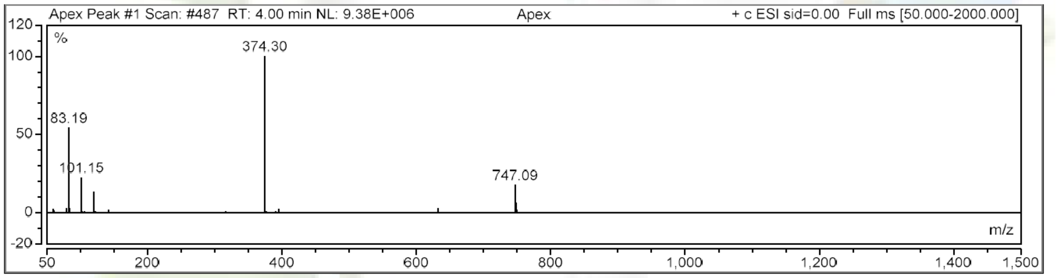

Synthesis of Py-NTA-GFP

I. 1-Pyrenecarboxylic acid (457 mg, 1.85 mmol) was dissolved in 27 mL anhydrous dimethylformamide (DMF). EDC (354 mg, 1.85 mmol) and NHS (426 mg, 3.7 mmol) were added and the mixture was stirred for 1 h at 40°C using a magnetic stirrer. Triethylamine (TEA, 317 μL, 2.3 mmol) was added to adjust the pH to 8 and ensure a basic environment. Nα, Nα-Bis(carboxymethyl)-L-lysine hydrate (NTA, 520 mg, 1.85 mmol) was added to the reaction mixture, followed by stirring for 72 h at 60°C. For purification, the reaction mixture was dissolved in 50 mL water and titrated with hydrochloric acid (HCl, 1 mol/L). Finally, 2,2'-((1-carboxy-5-(pyrene-1-carboxamido)pentyl)azanediyl)diacetic acid (Py-NTA) was obtained after centrifugation and lyophilization (16.4 mg, 44%).

II. Py-NTA (9.8 mg, 0.02 mmol) and $NiCl_2$ (2.6 mg, 0.02 mmol) were dissolved in 5 mL water, respectively. A syringe pump was used to add $NiCl_2$ solution to Py-NTA solution (10 mL/h), followed by stirring for 2 h at room temperature. Py-NTA-Ni^{2+} was obtained by repeated washing by centrifuge (14000 rpm, 10 min). The supernatants were destructed by 1% nitric acid overnight and the concentration of Ni^{2+} was detected by ICP-MS (n = 3).

III. 5 µL GFP solution (1500 µg/mL in PBS) was added to 1 mL of Py-NTA-Ni^{2+} solution (2 mM in Milli-Q water) under vigorous stirring at room temperature and the mixture was stirred overnight.

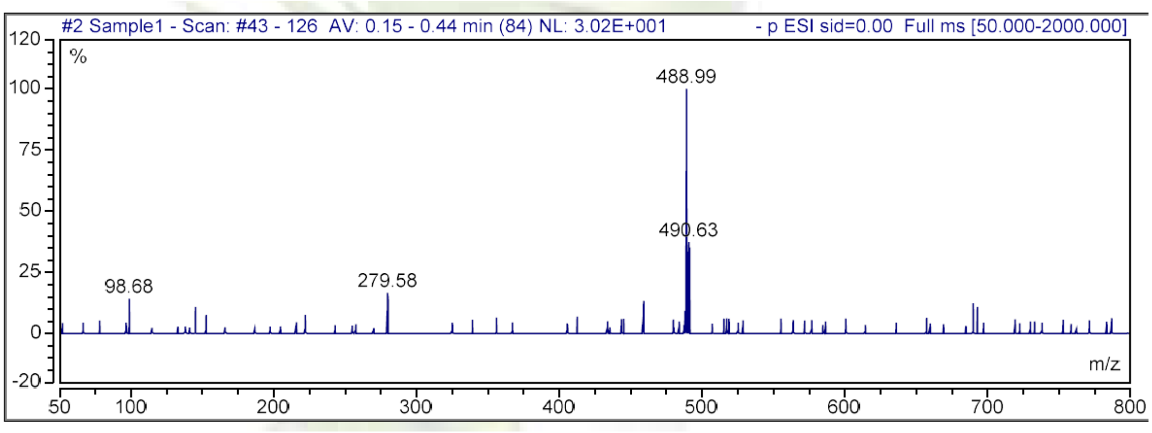
#2 Sample1 - Scan: #43 - 126 AV: 0.15 - 0.44 min (84) NL: 3.02E+001
- p ESI sid=0.00 Full ms [50.000-2000.000]
%
488.99
490.63
98.68
279.58
m/z

Supplementary Figures

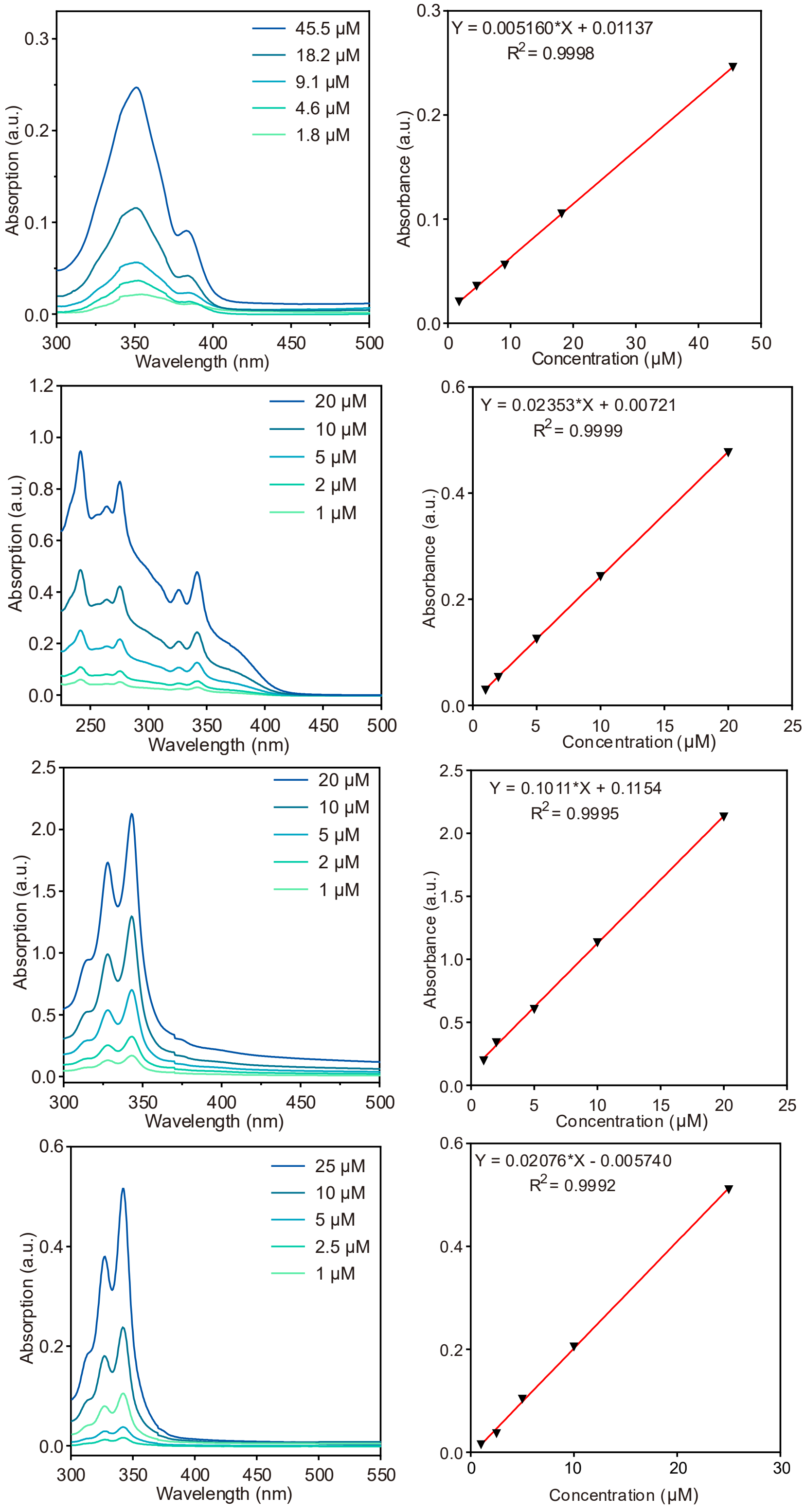

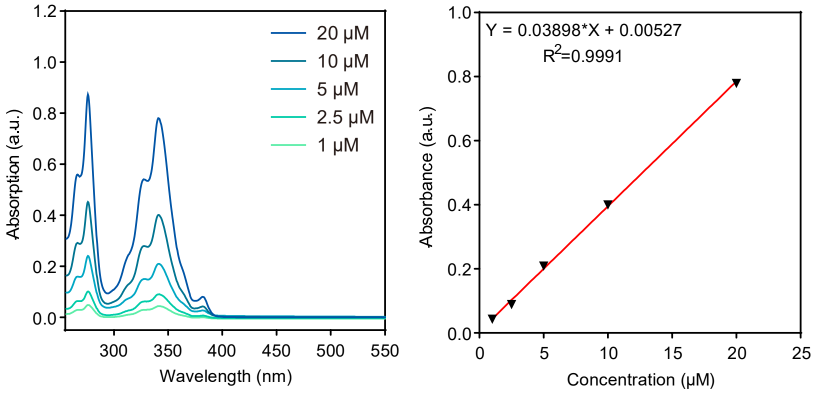

Figure S1. UV spectrums and linear calibration curves of Py-Xs. From top to bottom: Py-TPP, Py-FA, Py-HA, Py-RGD, and Py-NTA.

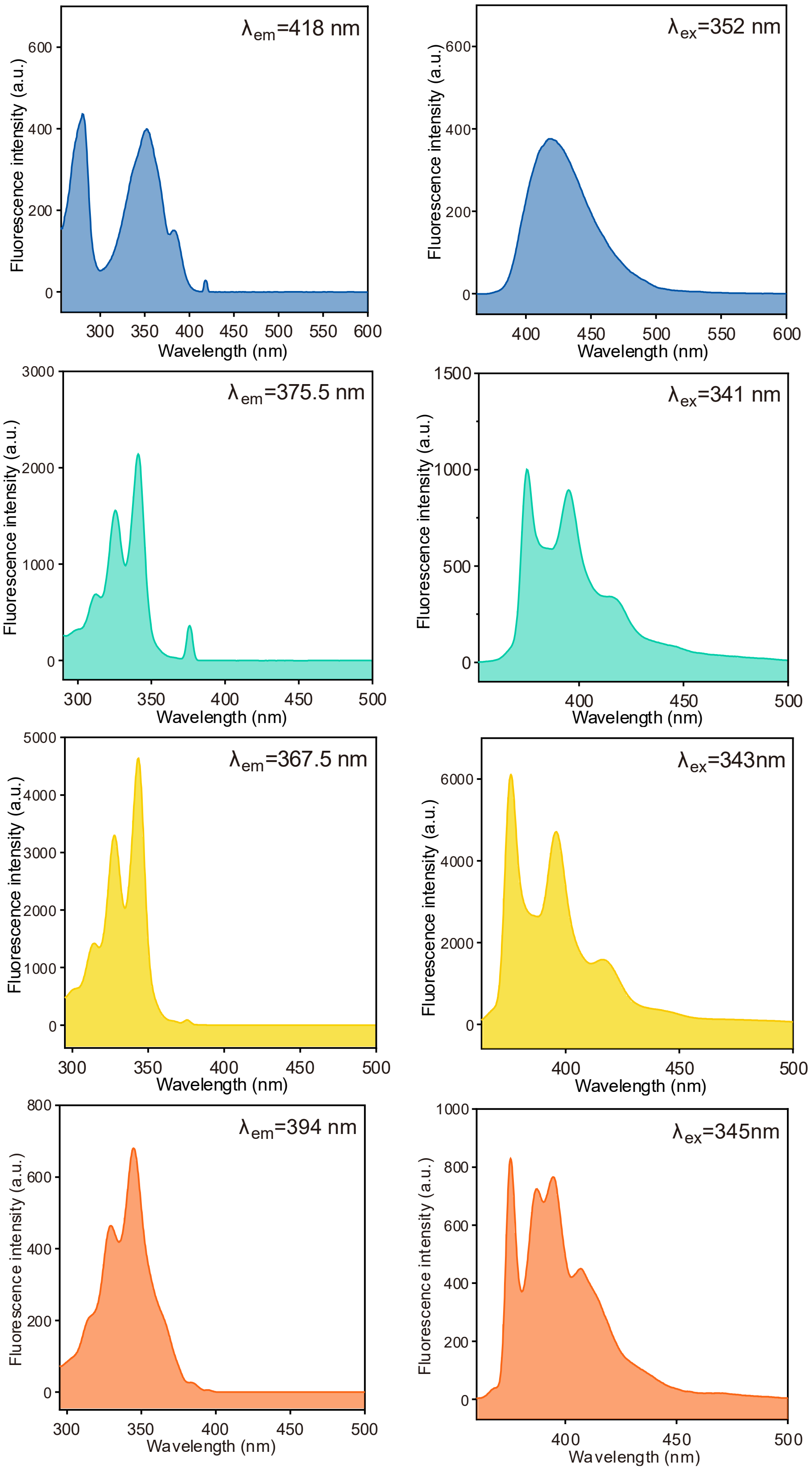
λem=418 nm
λex=352 nm
λem=375.5 nm
λex=341 nm
λem=367.5 nm
λex=343nm
λem=394 nm
λex=345nm
Fluorescence intensity (a.u.)
Wavelength (nm)

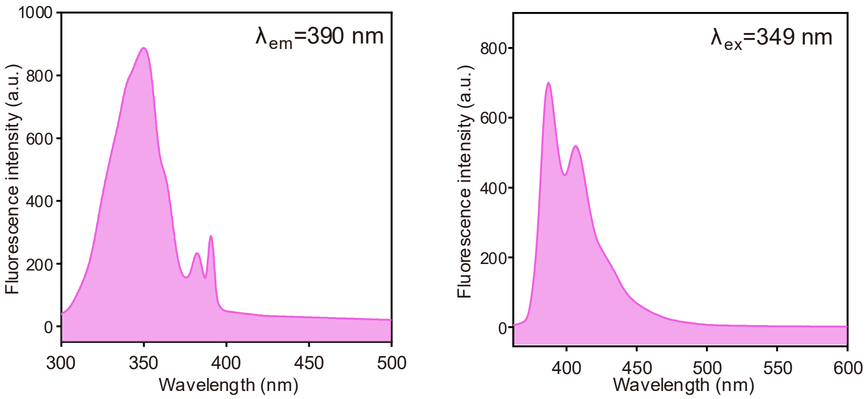

Figure S2. Fluorescence excitation spectrums and emission spectrums of Py-Xs. From top to bottom: Py-TPP, Py-FA, Py-HA, Py-RGD, and Py-NTA.

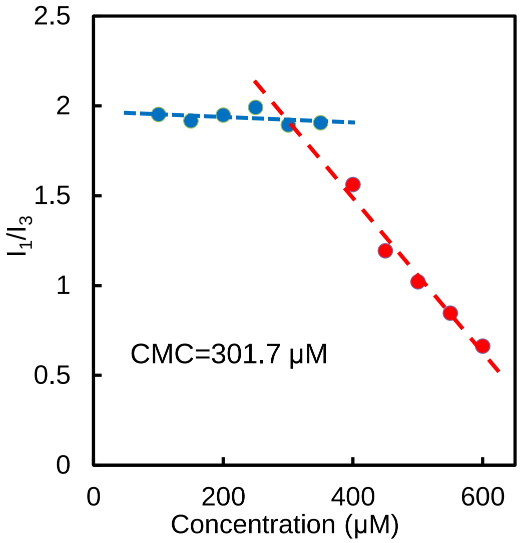

2.5
2
1.5
1
0.5
0
I_1/I_3
CMC=301.7 μM
0
200
400
600
Concentration (μM)

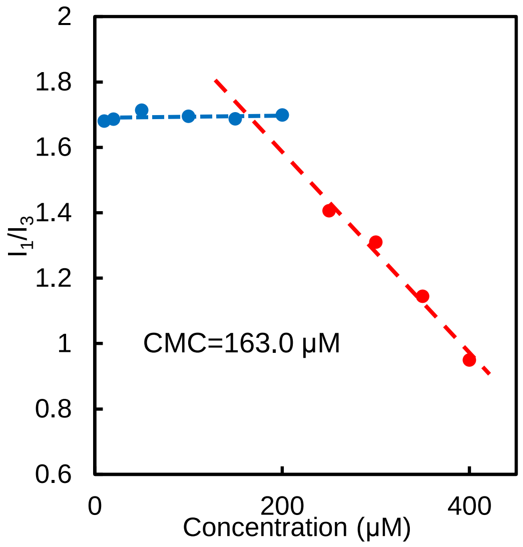

2
1.8
1.6
1.4
1.2
1
0.8
0.6
I_1/I_3
CMC=163.0 μM
0
200
400
Concentration (μM)

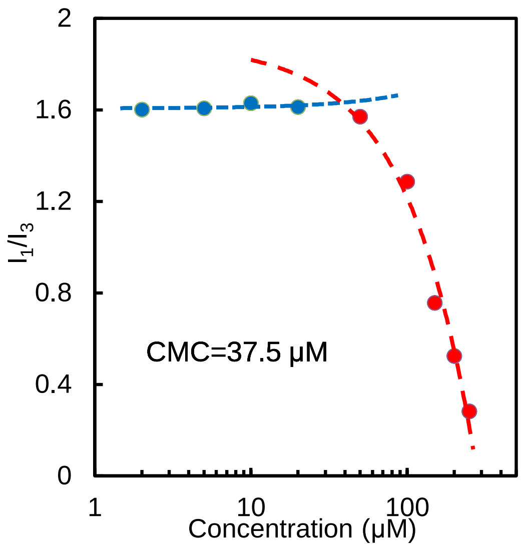
2
1.6
1.2
0.8
0.4
0
I_1/I_3
CMC=37.5 μM
1
10
100
Concentration (μM)

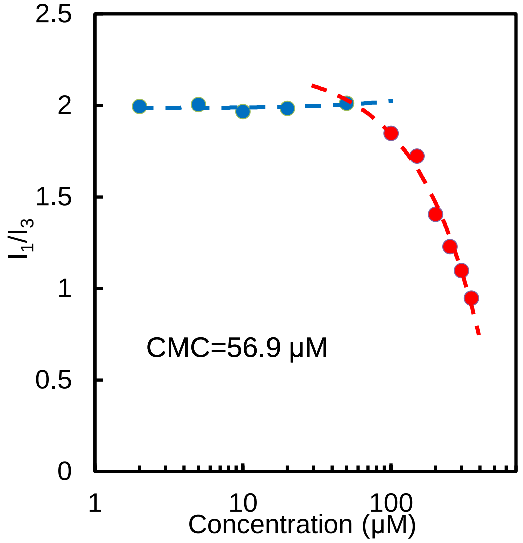
2.5
2
1.5
1
0.5
0
I_1/I_3
CMC=56.9 μM
1
10
100
Concentration (μM)

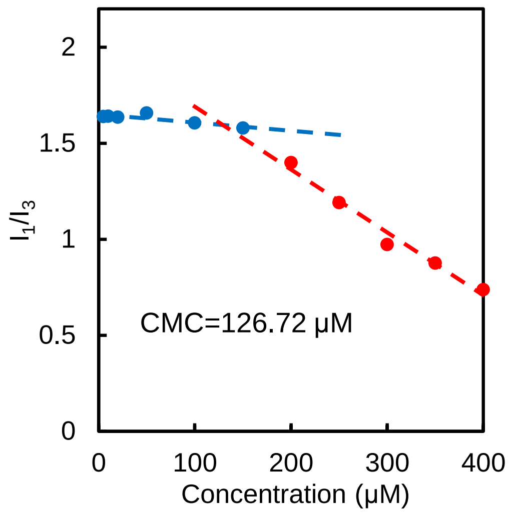

Figure S3. Critical micelle concentration (CMC) of Py-Xs. From top to bottom: Py-TPP, Py-FA, Py-HA, Py-RGD, and Py-NTA.

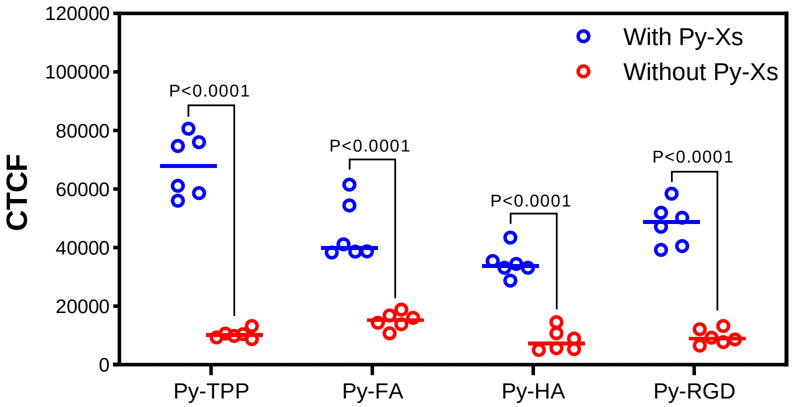

Figure S4. Fluorescence intensity analysis of micrometer-sized polymersomes. Significant differences were found in the CTCF before and after the insertion of each molecule.

Py-FA, Hela^{UFR}:

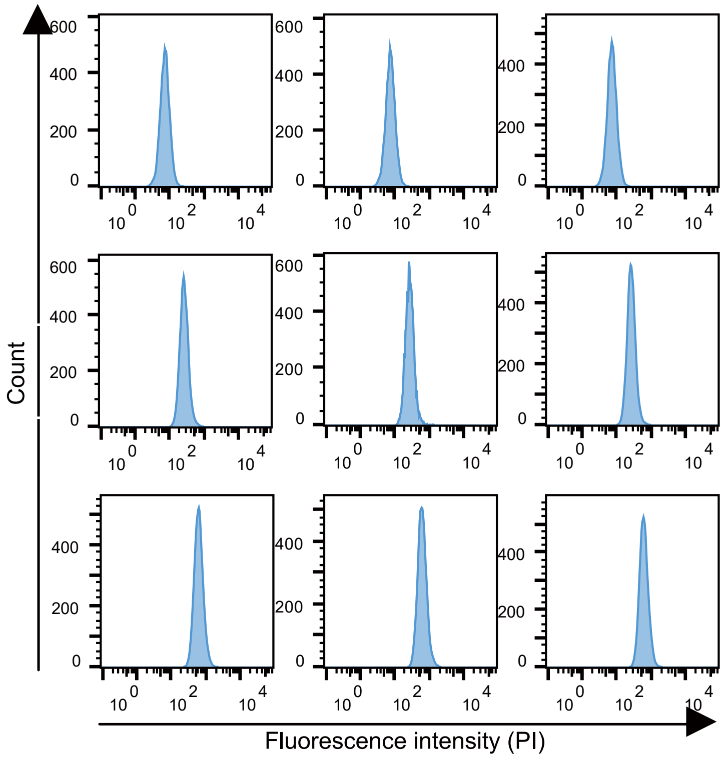

Py-FA, Hela^{-}:

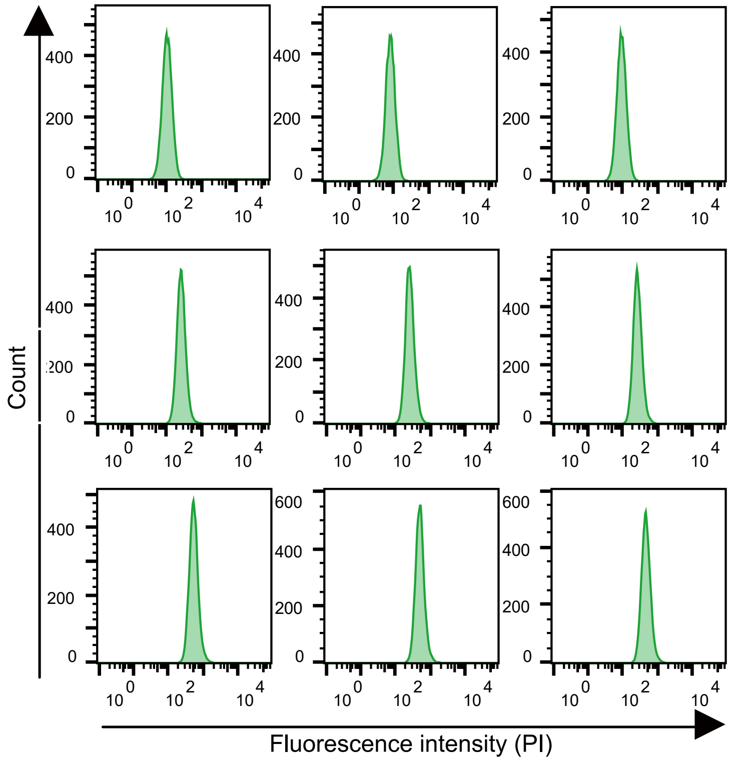

Py-Fa, NIH-3T3:

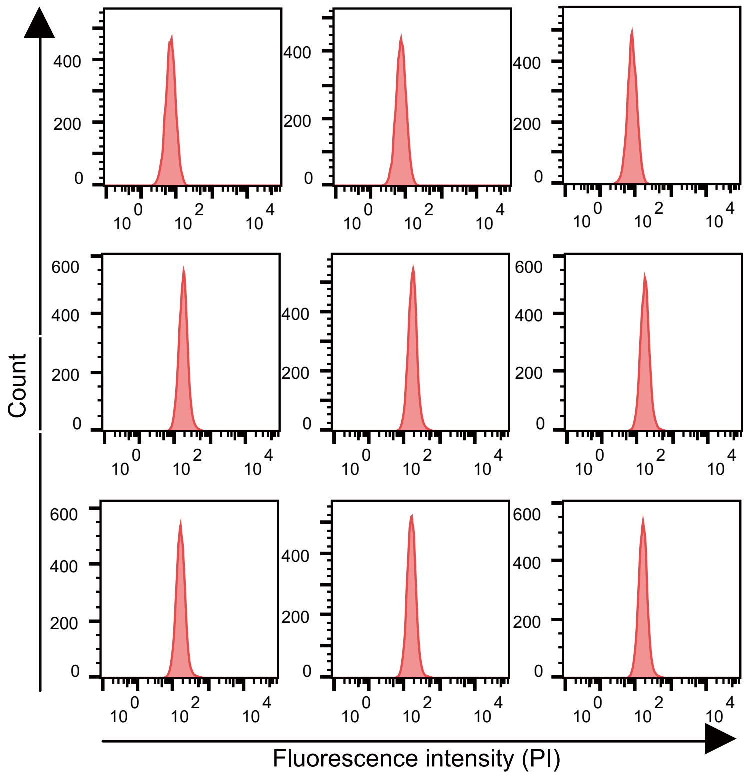

Py-RGD, Hela:

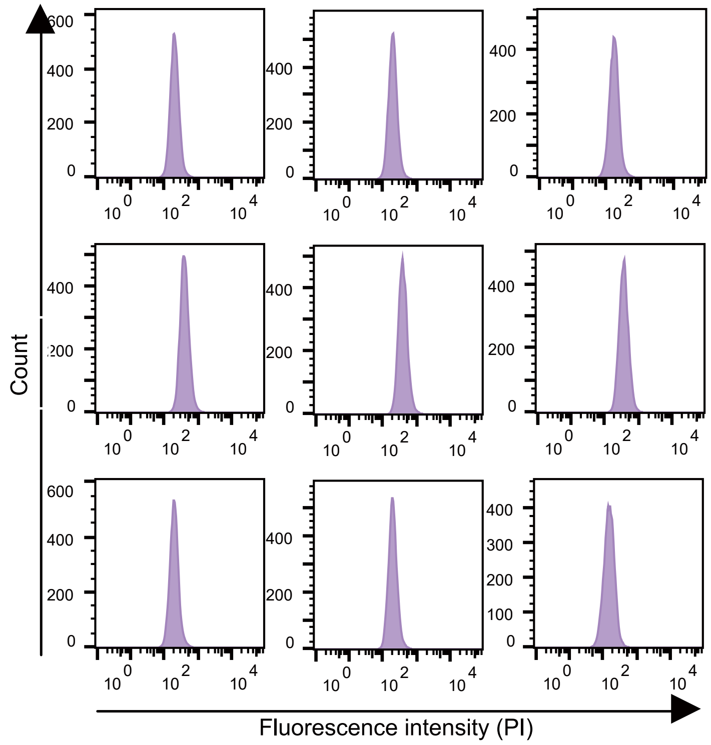

Figure S5. Individual flow cytometry data presented in Figure 5.

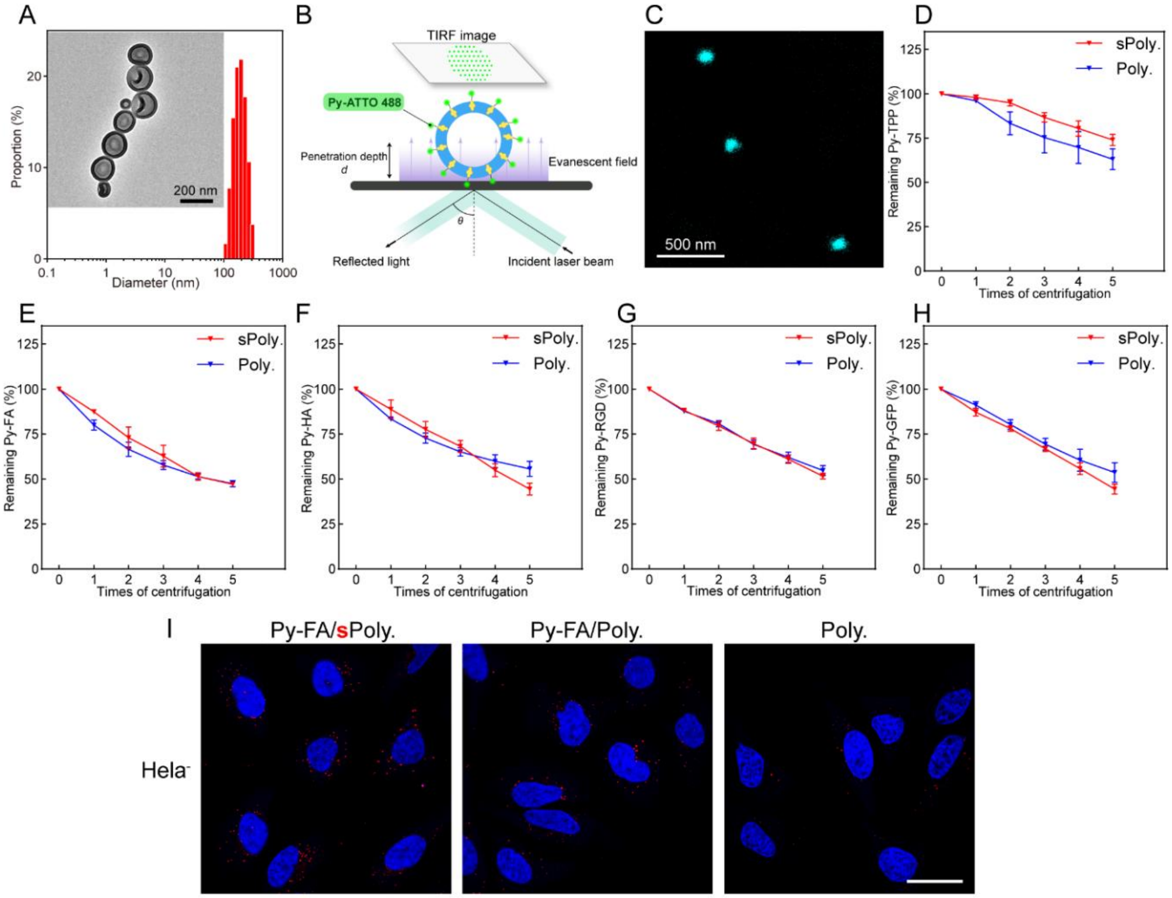

Figure S6. Insertion of Py-Xs onto small polymersomes (sPoly). (A) TEM image and size distribution of sPoly. (B) Illustration of the dStorm microscope in combination with internal reflection fluorescence (TIRF) mode for the visualization of Py-ATTO. (C) Super-resolution image of sPoly inserted by Py-ATTO. (D)(E)(F)(G) and (H) Stability test of Py-Xs loading onto sPoly in phosphate buffer (PBS, pH 7.2). Centrifugation: 14000 rpm, 10 min. (I) fluorescence images showing Hela^{-} incubated with Py-FA functionalized sPoly; scale bars = 20 μm.

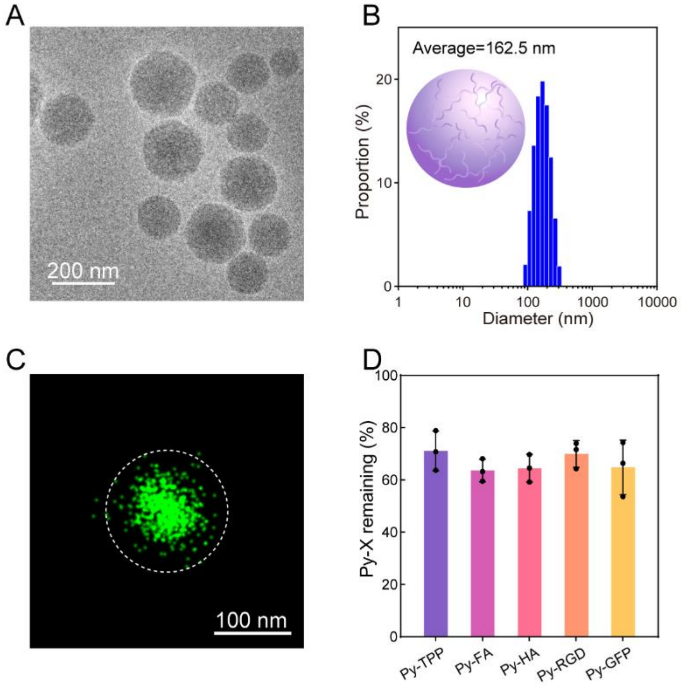

Figures S7. Insertion validation on PEG nanogels. (A) Cyro-TEM image of the PEG nanogels. (B) Size distribution of the PEG nanogels. (C) Super-resolution image of PEG nanogels inserted by Py-ATTO. (D)Remaining Py-Xs on PEG nanogels after three rounds of centrifugation in PBS (pH 7.2).

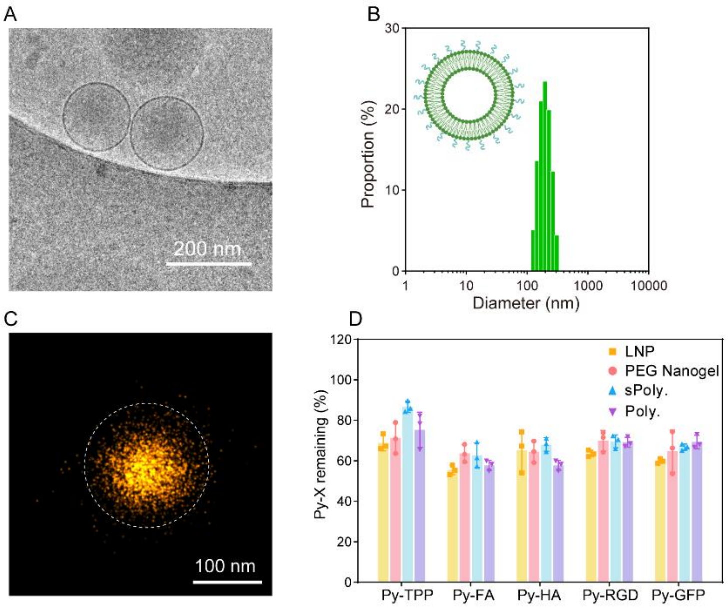

Figures S8. Insertion validation on lipid nanoparticles. (A) Cyro-TEM image of the liposomes. (B) Size distribution of the liposomes measured by DLS. (C) Super-resolution image of liposomes inserted by Py-ATTO. (D) Remaining Py-Xs on liposomes after three rounds of centrifugation in PBS (pH 7.2).

Supplementary Tables

Table S1. Zeta-potential of polymersomes before and after the insertion of Py-Xs

Entry	Before insertion			After insertion			*p* value
	Z-potential (mV)	Mean	SD	Z-potential (mV)	Mean	SD	
**Py-TPP**	-32.60<br>-33.40<br>-34.00	-33.33	0.70	24.80<br>27.00<br>26.30	26.03	1.12	< 0.0001
**Py-FA**	-31.60<br>-31.00<br>-32.20	-31.60	0.60	-39.30<br>-39.40<br>-39.00	-39.23	0.21	< 0.0001
**Py-HA**	-30.20<br>-30.50<br>-30.10	-30.27	0.21	-30.00<br>-29.80<br>-30.40	-30.10	0.31	0.4018
**Py-RGD**	-30.00<br>-29.20<br>-29.80	-29.67	0.42	-34.90<br>-35.60<br>-34.40	-34.97	0.60	< 0.0001
**Py-GFP**	-31.30<br>-31.30<br>-31.00	-31.20	0.17	-33.70<br>-33.30<br>-34.10	-33.70	0.40	0.0006

Table S2. Quantitative calculation of the number of Py-Xs loaded onto polymersomes upon centrifugation.

**Times of centrifugation**	**1**	**2**	**3**	**4**	**5**
Py-TPP inserted (%)	96.05 ± 2.26	84.80 ± 1.60	70.72 ± 1.62	61.80 ± 3.01	45.03 ± 3.59
Py-TPP inserted (uM)	19.21 ± 0.45	16.96 ± 0.32	14.14 ± 0.32	12.36 ± 0.60	9.01 ± 0.72
Py-TPP inserted (N_A=6.02×10^{23})	(1.16±0.03)×10^{16}	(1.02±0.02)×10^{16}	(0.85±0.02)×10^{16}	(0.74±0.04)×10^{16}	(0.54±0.04)×10^{16}
Py-TPP per polymersome	(2.17±0.05)×10^{4}	(1.95±0.04)×10^{4}	(1.77±0.04)×10^{4}	(1.62±0.08)×10^{4}	(1.18±0.09)×10^{4}

**Times of centrifugation**	**1**	**2**	**3**	**4**	**5**
Py-FA inserted (%)	69.99 ± 2.36	53.47 ± 1.90	44.23 ± 3.66	41.89 ± 3.98	34.54 ± 2.14
Py-FA inserted (uM)	14.00 ± 0.47	10.69 ± 0.38	8.85 ± 0.73	8.38 ± 0.80	6.91 ± 0.43
Py-FA inserted (N_A=6.02×10^{23})	(8.43±0.29)×10^{15}	(6.44±0.24)×10^{15}	(5.33±0.44)×10^{15}	(5.05±0.48)×10^{15}	(4.16±0.26)×10^{15}
Py-FA per polymersome	(1.58±0.05)×10^{4}	(1.23±0.04)×10^{4}	(1.11±0.09)×10^{4}	(1.10±0.10)×10^{4}	(0.90±0.6)×10^{4}

**Times of centrifugation**	**1**	**2**	**3**	**4**	**5**
Py-HA inserted (%, cal. as Py)	81.82 ± 0.76	74.63 ± 2.69	67.55 ± 3.53	61.72 ± 3.63	60.36 ± 4.20
Py-HA inserted (uM, cal. as Py)	16.36 ± 0.15	14.93 ± 0.54	13.51 ± 0.71	12.34 ± 0.73	12.07 ± 0.84
Py-HA inserted (uM)	1.26 ± 0.01	1.15 ± 0.04	1.04 ± 0.05	0.95 ± 0.06	0.93 ±0.06
Py-HA inserted (N_A=6.02×10^{23})	(7.58±0.07)×10^{14}	(6.91±0.25)×10^{14}	(6.26±0.33)×10^{14}	(5.72±0.34)×10^{14}	(5.71±0.47)×10^{14}
Py-HA per polymersome	(1.41±0.01)×10^{3}	(1.32±0.05)×10^{3}	(1.26±0.13)×10^{3}	(1.24±0.73)×10^{3}	(1.24±0.10)×10^{3}

**Times of centrifugation**	**1**	**2**	**3**	**4**	**5**
Py-RGD inserted (%)	88.23 ± 2.96	75.59 ± 6.10	63.98 ± 3.45	57.39 ± 3.40	45.94 ± 4.80
Py-RGD inserted (uM)	17.65 ± 0.59	15.12 ± 1.22	12.80 ± 0.69	11.48 ± 0.68	9.19 ± 0.96
Py-RGD inserted (N_A=6.02×10^{23})	(1.06±0.04)×10^{16}	(0.91±0.07)×10^{16}	(0.77±0.04)×10^{16}	(0.69±0.04)×10^{16}	(0.55±0.06)×10^{16}

Py-RGD per polymersome	$(1.99±0.07)×10^{4}$	$(1.74±0.14)×10^{4}$	$(1.61±0.09)×10^{4}$	$(1.50±0.09)×10^{4}$	$(1.20±0.13)×10^{4}$

**Times of centrifugation**	**1**	**2**	**3**	**4**	**5**
Py-GFP inserted (%)	87.78 ± 2.23	76.06 ± 2.93	63.26 ± 2.35	52.17 ± 1.98	45.45 ± 1.61
Py-GFP inserted (uM)	9.11 ± 0.19	8.03 ± 0.28	6.93 ± 0.33	6.05 ± 0.61	5.36 ± 0.54
Py-GFP inserted (N_A=6.02×10^{23})	$(5.29±0.13)×10^{15}$	$(4.58±0.18)×10^{15}$	$(3.81±0.14)×10^{15}$	$(3.14±0.12)×10^{15}$	$(2.74±0.10)×10^{15}$
Py-GFP per polymersome	$(9.90±0.25)×10^{3}$	$(8.74±0.34)×10^{3}$	$(7.94±0.30)×10^{3}$	$(6.83±0.26)×10^{3}$	$(5.94±0.21)×10^{3}$

Table S3. Stability of Py-Xs in complete cell culture medium

Entry	Initial concentration (μM)	Py-Xs inserted (μM)	Py-Xs inserted (%, mean±SD)
Py-TPP	20	14.41<br>13.95<br>15.39	72.91±3.67
Py-FA	20	10.75<br>11.23<br>12.02	56.67±3.21
Py-HA	20	13.27<br>13.22<br>12.11	64.32±3.29
Py-RGD	20	12.55<br>10.28<br>12.36	58.65±6.28
Py-GFP	10	6.14<br>5.72<br>5.85	59.05±2.11

Legend for data S1: Raw data points in Supplementary Information.

REFERENCES AND NOTES

1. J. J. Shi, A. R. Votruba, O. C. Farokhzad, R. Langer, Nanotechnology in drug delivery and tissue engineering: From discovery to applications. *Nano Lett.* **10**, 3223–3230 (2010).

2. A. Singh, M. M. Amiji, Application of nanotechnology in medical diagnosis and imaging. *Curr. Opin. Biotech.* **74**, 241–246 (2022).

3. L. J. Peek, C. R. Middaugh, C. Berkland, Nanotechnology in vaccine delivery. *Adv. Drug Deliver. Rev.* **60**, 915–928 (2008).

4. C. J. Kearney, D. J. Mooney, Macroscale delivery systems for molecular and cellular payloads. *Nat. Mater.* **12**, 1004–1017 (2013).

5. A. Akinc, M. A. Maier, M. Manoharan, K. Fitzgerald, M. Jayaraman, S. Barros, S. Ansell, X. Y. Du, M. J. Hope, T. D. Madden, B. L. Mui, S. C. Semple, Y. K. Tam, M. Ciufolini, D. Witzigmann, J. A. Kulkarni, R. van der Meel, P. R. Cullis, The Onpattro story and the clinical translation of nanomedicines containing nucleic acid-based drugs. *Nat. Nanotechnol.* **14**, 1084–1087 (2019).

6. P. V. Pawar, S. V. Gohil, J. P. Jain, N. Kumar, Functionalized polymersomes for biomedical applications. *Polym. Chem. Uk.* **4**, 3160–3176 (2013).

7. R. Mout, D. F. Moyano, S. Rana, V. M. Rotello, Surface functionalization of nanoparticles for nanomedicine. *Chem. Soc. Rev* **41**, 2539–2544 (2012).

8. H. C. Kolb, M. G. Finn, K. B. Sharpless, Click chemistry: Diverse chemical function from a few good reactions. *Angew Chem Int Ed. Engl.* **40**, 2004 (2001).

9. S. Egli, H. Schlaad, N. Bruns, W. Meier, Functionalization of block copolymer vesicle surfaces. *Polymers Basel.* **3**, 252–280 (2011).

10. K. E. Sapsford, W. R. Algar, L. Berti, K. B. Gemmill, B. J. Casey, E. Oh, M. H. Stewart, I. L. Medintz, Functionalizing nanoparticles with biological molecules: Developing chemistries that facilitate nanotechnology. *Chem. Rev.* **113**, 1904–2074 (2013).

11. J. B. Luan, D. Wang, S. Zhang, Y. Miyazaki, W. Shinoda, D. A. Wilson, Complex energy landscapes of self-assembled vesicles. *J. Am. Chem. Soc.* **145**, 15496–15506 (2023).

12. L. Y. Xu, X. N. Yang, Molecular dynamics simulation of adsorption of pyrene-polyethylene glycol onto graphene. *J. Colloid Interf. Sci.* **418**, 66–73 (2014).

13. U. R. Dahal, E. E. Dormidontova, Spontaneous insertion, helix formation, and hydration of polyethylene oxide in carbon nanotubes. *Phys. Rev. Lett.* **117**, 027801 (2016).

14. S. H. Zhang, W. Li, J. B. Luan, A. Srivastava, V. Carnevale, M. L. Klein, J. W. Sun, D. N. Wang, S. P. Teora, S. J. Rijpkema, J. D. Meeldijk, D. A. Wilson, Adaptive insertion of a hydrophobic anchor into a poly(ethylene glycol) host for programmable surface functionalization. *Nat. Chem.* **15**, 240–247 (2023).

15. S. H. Zhang, A. Srivastava, W. Li, S. J. Rijpkema, V. Carnevale, M. L. Klein, D. A. Wilson, Molecular engineering of pH-responsive anchoring systems onto poly(ethylene glycol) corona. *J. Am. Chem. Soc.* **145**, 10458–10462 (2023).

16. A. K. Chaudhary, N. Yadav, T. A. Bhat, J. O'Malley, S. Kumar, D. Chandra, A potential role of X-linked inhibitor of apoptosis protein in mitochondrial membrane permeabilization and its implication in cancer therapy. *Drug Discov. Today* **21**, 38–47 (2016).

17. J. S. Modica-Napolitano, K. K. Singh, Mitochondrial dysfunction in cancer. *Mitochondrion* **4**, 755–762 (2004).

18. L. Galluzzi, E. Morselli, O. Kepp, I. Vitale, A. Rigoni, E. Vacchelli, M. Michaud, H. Zischka, M. Castedo, G. Kroemer, Mitochondrial gateways to cancer. *Mol. Aspects Med.* **31**, 1–20 (2010).

19. Z. J. Wang, W. L. Guo, X. Kuang, S. S. Hou, H. Z. Liu, Nanopreparations for mitochondria targeting drug delivery system: Current strategies and future prospective. *Asian J. Pharm. Sci.* **12**, 498–508 (2017).

20. J. R. Friedman, J. Nunnari, Mitochondrial form and function. *Nature* **505**, 335–343 (2014).

21. J. S. Modica-Napolitano, J. R. Aprille, Delocalized lipophilic cations selectively target the mitochondria of carcinoma cells. *Adv. Drug Deliver Rev.* **49**, 63–70 (2001).

22. J. Sudimack, R. J. Lee, Targeted drug delivery via the folate receptor. *Adv. Drug Deliver Rev.* **41**, 147–162 (2000).

23. S. Sabharanjak, S. Mayor, Folate receptor endocytosis and trafficking. *Adv. Drug Deliver Rev.* **56**, 1099–1109 (2004).

24. L. Y. W. Bourguignon, Hyaluronan-mediated CD44 activation of RhoGTPase signaling and cytoskeleton function promotes tumor progression. *Semin. Cancer Biol.* **18**, 251–259 (2008).

25. G. Mattheolabakis, L. Milane, A. Singh, M. M. Amiji, Hyaluronic acid targeting of CD44 for cancer therapy: From receptor biology to nanomedicine. *J. Drug Target.* **23**, 605–618 (2015).

26. Y. Yamauchi, A. Helenius, Virus entry at a glance. *J. Cell Sci.* **126**, 1289–1295 (2013).

27. A. M. Alkilany, L. Zhu, H. Weller, A. Mews, W. J. Parak, M. Barz, N. Feliu, Ligand density on nanoparticles: A parameter with critical impact on nanomedicine. *Adv. Drug Deliver Rev.* **143**, 22–36 (2019).

28. M. A. Hink, R. A. Griep, J. W. Borst, A. van Hoek, M. H. M. Eppink, A. Schots, A. J. W. G. Visser, Structural dynamics of green fluorescent protein alone and fused with a single chain Fv protein. *J. Biol. Chem.* **275**, 17556–17560 (2000).

29. J. F. Guo, M. Schlich, J. F. Cryan, C. M. O'Driscoll, Targeted drug delivery via folate receptors for the treatment of brain cancer: Can the promise deliver? *J. Pharm. Sci.* **106**, 3413–3420 (2017).

30. M. A. Kane, P. C. Elwood, R. M. Portillo, A. C. Antony, V. Najfeld, A. Finley, S. Waxman, J. F. Kolhouse, Influence on immunoreactive folate-binding proteins of extracellular folate concentration in cultured human cells. *J. Clin. Invest* **81**, 1398–1406 (1988).

31. D. Feng, Y. C. Song, W. Shi, X. H. Li, H. M. Ma, Distinguishing folate-receptor-positive cells from folate-receptor-negative cells using a fluorescence off-on nanoprobe. *Anal Chem.* **85**, 6530–6535 (2013).

32. P. G. Hu, L. M. Chen, X. W. Kang, S. W. Chen, Surface functionalization of metal nanoparticles by conjugated metal-ligand interfacial bonds: Impacts on intraparticle charge transfer. *Accounts Chem. Res.* **49**, 2251–2260 (2016).

33. A. Lamoot, A. Uvyn, S. Kasmi, B. G. De Geest, Covalent cell surface conjugation of nanoparticles by a combination of metabolic labeling and click chemistry. *Angew. Chem. Int. Ed. Engl.* **60**, 6320–6325 (2021).

34. G. Sanità, B. Carrese, A. Lamberti, Nanoparticle surface functionalization: How to improve biocompatibility and cellular internalization. *Front. Mol. Biosci.* **7**, 587012 (2020).

35. N. Boehnke, K. J. Dolph, V. M. Juarez, J. M. Lanoha, P. T. Hammond, Electrostatic conjugation of nanoparticle surfaces with functional peptide motifs. *Bioconjugate Chem.* **31**, 2211–2219 (2020).

36. T. Thomsen, R. Reissmann, E. Kaba, B. Engelhardt, H. A. Klok, Covalent and noncovalent conjugation of degradable polymer nanoparticles to T lymphocytes. *Biomacromolecules* **22**, 3416–3430 (2021).

37. T. G. Papaioannou, C. Stefanadis, Vascular wall shear stress: Basic principles and methods. *Hellenic J. Cardiol.* **46**, 9–15 (2005).

38. J. Sun, M. Mathesh, W. Li, D. A. Wilson, Enzyme-powered nanomotors with controlled size for biomedical applications. *ACS Nano* **13**, 10191–10200 (2019).

39. M. Y. Hu, Y. Wang, Z. S. Liu, Z. Yu, K. Y. Guan, M. R. Liu, M. L. Wang, J. Tan, L. A. Huang, Hepatic macrophages act as a central hub for relaxin-mediated alleviation of liver fibrosis. *Nat. Nanotechnol.* **16**, 466–477 (2021).

40. M. C. Sun, X. L. Xu, Y. Du, X. F. Lou, W. Wang, Y. C. You, D. Liu, F. Y. Jin, J. Qi, M. X. Zhu, L. W. Zhu, J. Wang, Y. Z. Du, Biomimetic melanosomes promote orientation-selective delivery and melanocyte pigmentation in the HO-induced vitiligo mouse model. *ACS Nano* **15**, 17361–17374 (2021).
